# Effectors of the Stenotrophomonas maltophilia Type IV Secretion System Mediate Killing of Clinical Isolates of Pseudomonas aeruginosa

**DOI:** 10.1128/mBio.01502-21

**Published:** 2021-06-29

**Authors:** Megan Y. Nas, Jeffrey Gabell, Nicholas P. Cianciotto

**Affiliations:** a Department of Microbiology and Immunology, Northwestern University Medical School, Chicago, Illinois, USA; University of Michigan-Ann Arbor

**Keywords:** *Pseudomonas aeruginosa*, *Stenotrophomonas*, *Stenotrophomonas maltophilia*, bacterial competition, bactericidal activity, cystic fibrosis, lipase, peptidoglycan hydrolases, type IV secretion

## Abstract

Previously, we documented that Stenotrophomonas maltophilia encodes a type IV secretion system (T4SS) that allows the organism to kill, in contact-dependent fashion, heterologous bacteria, including wild-type Pseudomonas aeruginosa. Bioinformatic screens based largely on the presence of both a C-terminal consensus sequence and an adjacent gene encoding a cognate immunity protein identified 13 potential antibacterial effectors, most of which were highly conserved among sequenced strains of S. maltophilia. The immunity proteins of two of these proved especially capable of protecting P. aeruginosa and Escherichia coli against attack from the *Stenotrophomonas* T4SS. In turn, S. maltophilia mutants lacking the putative effectors RS14245 and RS14255 were impaired for killing not only laboratory E. coli but clinical isolates of P. aeruginosa, including ones isolated from the lungs of cystic fibrosis patients. That complemented mutants behaved as wild type did confirmed that RS14245 and RS14255 are required for the bactericidal activity of the S. maltophilia T4SS. Moreover, a mutant lacking both of these proteins was as impaired as a mutant lacking the T4SS apparatus, indicating that RS14245 and RS14255 account for (nearly) all of the bactericidal effects seen. Utilizing an interbacterial protein translocation assay, we determined that RS14245 and RS14255 are bona fide substrates of the T4SS, a result confirmed by examination of mutants lacking both the T4SS and the individual effectors. Delivery of the cloned 14245 protein (alone) into the periplasm resulted in the killing of target bacteria, indicating that this effector, a putative lipase, is both necessary and sufficient for bactericidal activity.

## INTRODUCTION

Gram-negative Stenotrophomonas maltophilia is a frequent isolate from water, soil, and plant material and the most characterized member of the *Stenotrophomonas* genus, which currently consists of 19 species (1, 2). S. maltophilia is also an increasingly important pathogen particularly in the hospital setting but also in the broader community (3–6). S. maltophilia infection is most often manifest as pneumonia and bacteremia (4, 7, 8). The bacterium is also notable in cystic fibrosis (CF) patients, where S. maltophilia infection heightens the risk of lung exacerbations (9–13). Very recent studies indicate that S. maltophilia often infects COVID-19 patients, suggesting that its importance is growing more pronounced (14–16). Many strains of S. maltophilia have resistance to a range of antibiotics, explaining, to a large extent, the clinical problem that is S. maltophilia (17–19). We and others have shown that S. maltophilia has many traits linked to virulence, including growth in the lungs of infected mice, attachment to host cells, secretion of degradative enzymes via a type II secretion system, siderophore production, and biofilm formation (20–30). Moreover, we recently documented that S. maltophilia encodes a type IV secretion system (T4SS) that has a profound role in the interactions of S. maltophilia with mammalian host cells and other types of bacteria (31).

Spanning the Gram-negative cell wall, the T4SS apparatus consists of 12 core proteins (32). Cytoplasm-facing VirD4 is a coupling protein for the recruitment of substrates to an inner membrane complex made of VirB3, VirB6, VirB8, VirB4, and VirB11. Substrates are then secreted out through a periplasm-outer membrane-spanning complex composed of VirB7, VirB9, and VirB10. VirB2 and VirB5 form a pilus-like structure designed to contact target membranes, and VirB1 helps degrade peptidoglycan during assembly of the T4SS apparatus. T4SSs are important in environmental species of *Agrobacterium*, *Lysinibacillus*, *Piscirickettsia*, *Sinorhizobium*, *Wolbachia*, and *Xanthomonas*, promoting various interactions with fish, plants, insects, and other microbes (33–41). T4SSs also play a major role for human and other mammal pathogens, including species of *Actinobacillus*, *Anaplasma*, *Bartonella*, *Bordetella*, Brucella, *Burkholderia*, *Coxiella*, *Ehrlichia*, *Helicobacter*, *Legionella*, *Neisseria*, and *Rickettsia* (42–57). We recently showed that environmental and clinical isolates of S. maltophilia encode a T4SS (31). This VirB/D4 T4SS is most similar to the T4SS of *Xanthomonas*, the genus most related to *Stenotrophomonas*. Based on analysis of a *virB10* mutant of clinical isolate K279a, along with a complemented derivative of that mutant, we showed that the S. maltophilia T4SS promotes the killing of human macrophages while blunting death in human epithelial cells (31). Moreover, when we cocultured, for short periods of time, strain K279a with wild-type (WT) Pseudomonas aeruginosa, the T4SS reduced the numbers of the heterologous bacteria, signaling that some *Stenotrophomonas* effectors have bactericidal activity (31). The effects of the T4SS required contact with the target cell. We further observed that the S. maltophilia T4SS killed all three P. aeruginosa strains tested including environmental strain 7700 and clinical isolates PAO1 and PAK and that other strains of S. maltophilia also could kill P. aeruginosa (31). The S. maltophilia T4SS also promoted the death of Pseudomonas mendocina but not Pseudomonas fluorescens, Pseudomonas putida, or Pseudomonas stutzeri, implying that its bactericidal effect extends to multiple, but not all, Pseudomonas species (31). Our findings on the bactericidal effect of the S. maltophilia T4SS have been confirmed by others showing that strain K279a lyses target bacteria during short-term coincubation (58). In contrast to the vast amount of data on the antibacterial effects of type VI secretion systems (T6SSs) (59–64), knowledge of the antibacterial role of T4SSs is minimal, being limited to the two studies of S. maltophilia and other work on the plant pathogen Xanthomonas citri and animal pathogen Bartonella schoenbuchensis (31, 58, 65, 66). Moreover, the T4SS effectors necessary for the ability of S. maltophilia to kill wild-type strains of other species, including P. aeruginosa, have remained unknown. Here, we identified two bona fide substrates of the S. maltophilia T4SS (to be designated TfcA and TfcB) that promote the killing of clinical isolates of P. aeruginosa, including those from CF lungs. These data provide novel insight into role of T4SSs in competitions between human pathogens.

## RESULTS

### Identification and gene distribution and expression of putative antibacterial effectors of S. maltophilia T4SS.

**FIG 1:**
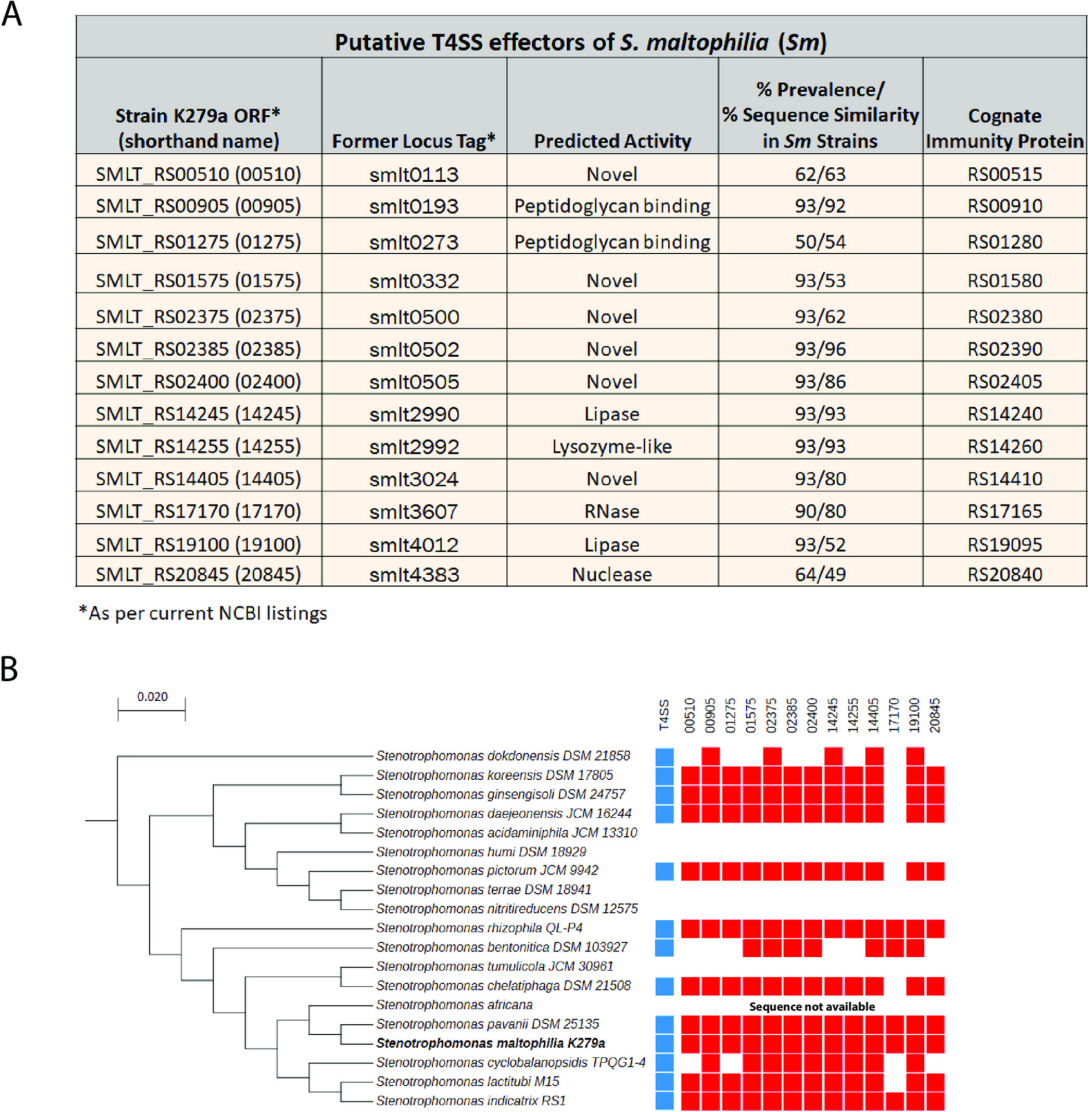
Putative T4SS effectors of S. maltophilia. (A) Thirteen putative effectors of strain K279a are presented, along with their current ORF designations and, in parentheses, the shorthand to be used throughout this paper (first column), former locus tags (second column), predicted activities based on annotations at NCBI (third column), percent prevalence among all of the sequenced strains of S. maltophilia/percent amino acid similarity of the proteins across the species (fourth column), and the current ORF designations for the putative cognate immunity proteins (fifth column). (B) Distribution of the putative effectors within the *Stenotrophomonas* genus. On the left side, the 19 named species of *Stenotrophomonas* are presented in a phylogenetic tree that is based on 16S rRNA sequences and scaled at 0.02 substitutions per site (2). On the right side, each column represents the distribution of one of the 13 effectors, with a red square denoting the presence of the corresponding gene and a blank denoting the absence of the gene. Also indicated in a similar manner is the presence (blue squares) or absence of the T4SS apparatus genes in the different species. No data are presented for Stenotrophomonas africana, since that species has not been entirely sequenced yet.

Using the T4SEpre and S4TE programs that are designed to recognize proteins that have C-terminal features (e.g., glutamic acid and serine-enriched, coiled versus helical) that are similar to those of known T4SS substrates from a variety of bacteria (67, 68), we performed an initial bioinformatics screen to identify potential antibacterial effectors of the T4SS of S. maltophilia strain K279a. After identifying proteins containing these general C-terminal features, we narrowed in on those that were annotated as having an activity that is compatible with antibacterial function. We then focused on a subset that were encoded adjacent to an open reading frame (ORF) for a putative immunity protein, since this type of gene arrangement commonly occurs for antibacterial effectors secreted by the T4SS of *X. citri* and T6SSs (65, 69, 70). This resulted in the identification of two putative effectors, namely, those encoded by ORFs 17170 and 19100 (Fig. 1A). As a second path to finding targets for investigation, we searched the K279a genome for proteins with C-terminal sequences that had more specific similarity to the C-terminal domains in *Xanthomonas* T4SS effectors that are implicated in binding to the VirD4 coupling protein (65, 71). This yielded nine proteins that are also encoded next to putative immunity proteins, with one of those being the 19100 protein identified in the first screen and the rest being encoded by ORFs 00905, 01575, 02375, 02385, 02400, 14245, 14255, and 14405 (Fig. 1A). As a third screen, we searched the K279a genome for homologs to proteins of other *Stenotrophomonas* strains that were previously found to be akin to *Xanthomonas* T4SS substrates (65). This resulted in both the reidentification of 00905, 02400, and 14255 and the discovery of the candidate encoded by ORF 20845 (Fig. 1A). Finally, we added K279a proteins from ORFs 00510 and 01275, which had recently been proposed as T4SS substrates, based on homologies to *Xanthomonas* proteins (58). Thus, we arrived at a list of 13 possible S. maltophilia T4SS substrates with activity against other bacteria (Fig. 1A). Two proteins were predicted to encode lipases, two were annotated as nucleases, and three appeared to interact with peptidoglycan (Fig. 1A); i.e., these seven were compatible with antibacterial function, as best exemplified by T6SS effectors (72). The remaining six did not show sequence similarity to known proteins, suggesting they might encode novel activities (Fig. 1A). Using BLAST, we determined that 10 of the putative effectors were encoded by 90 to 93% of the 42 sequenced strains of S. maltophilia (including K279a) (Fig. 1A and see Table S1A in the supplemental material). The strains that lacked the proteins also lacked genes for the T4SS apparatus. The remaining three putative substrates had a prevalence within the species between 50 and 64% (Fig. 1A and Table S1A). Thirteen of the strains had all 13 putative effectors, 10 others had 12, and eight others had 11 (Table S1A), suggesting that the repertoire of K279a is representative. The prevalence of the immunity proteins mirrored that of the putative effectors (Table S1B). All 13 of the putative effectors of S. maltophilia had homologs in other *Stenotrophomonas* species, with five of them occurring in all 13/13 species that carry a T4SS, and seven others being present in 11/13 or 10/13 of the T4SS^+^ species (Fig. 1B). From qRT-PCR analysis, we determined that all of the putative effector genes are expressed by S. maltophilia strain K279a grown on LB agar. Also, all were similarly expressed when the strain was grown in the presence of competing P. aeruginosa for 2 to 24 h (Fig. S1). Given that all of the putative effectors were both present in the majority of S. maltophilia strains and similarly expressed in the presence of a competitor, we considered all of them for mutational analysis.

10.1128/mBio.01502-21.1 FIG S1 Expression of transcripts encoding putative T4SS effectors of S. maltophilia. Wild-type strain K279a was incubated on LB agar at 37°C either alone or in the presence of P. aeruginosa strain 7700. After 2 h and 24 h of incubation, RNA was extracted and the levels of the 13 indicated S. maltophilia gene transcripts were determined by qRT-PCR. At each time point and for each gene examined, the transcript levels presented are normalized to what had occurred for strain K279a alone (dashed red line). Thus, all effector genes were expressed at each time point, with asterisks indicating those with differences between the value at 2 h and the value at 24 h (*, *P* < 0.05). Data are presented as the means and standard deviations of results from three independent experiments (*n* = 3 each). Download FIG S1, TIF file, 0.3 MB. Copyright © 2021 Nas et al. 2021 Nas et al. https://creativecommons.org/licenses/by/4.0/ This content is distributed under the terms of the Creative Commons Attribution 4.0 International license.

10.1128/mBio.01502-21.9 TABLE S1 (A) Presence of putative T4SS effector proteins in sequenced strains of S. maltophilia. (B) Presence of putative T4SS cognate immunity proteins in sequenced strains of S. maltophilia. Download Table S1, PDF file, 0.2 MB. Copyright © 2021 Nas et al. 2021 Nas et al. https://creativecommons.org/licenses/by/4.0/ This content is distributed under the terms of the Creative Commons Attribution 4.0 International license.

### Protective effects of S. maltophilia T4SS immunity proteins.

**FIG 2:**
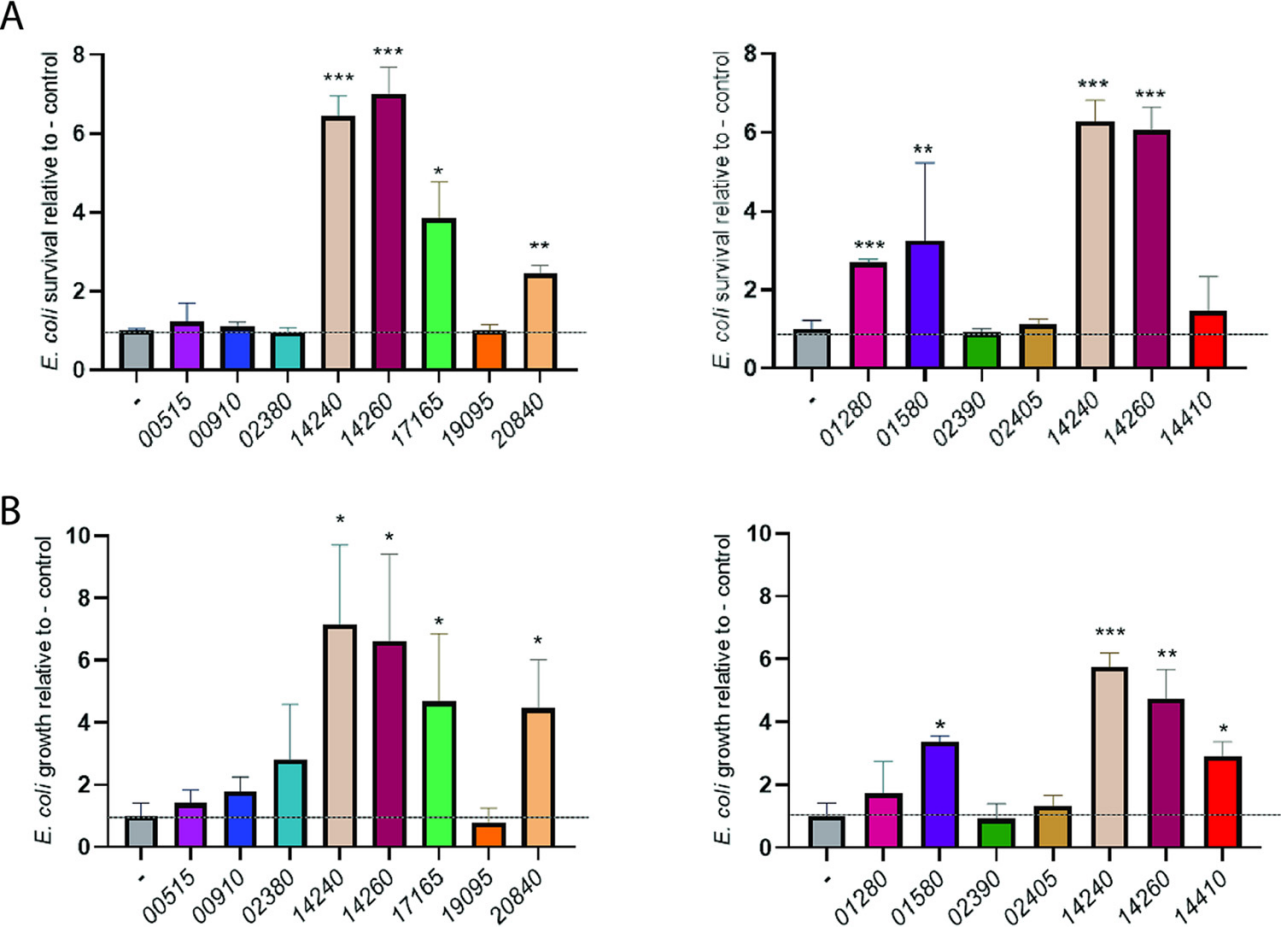
Ability of cloned immunity proteins to protect E. coli from killing by wild-type S. maltophilia. E. coli DH5α containing either the vector pBBR1MCS alone (-) or the cloned gene encoding one of the predicted immunity proteins from S. maltophilia K279a, i.e., 00515, 00910, 02380, 14240, 14260, 17165, 19095, or 20840 (left panels) or 01280, 01580, 02390, 02405, 14240, 14260, or 14410 (right panels), was mixed with S. maltophilia K279a in a (S. maltophilia/E. coli) ratio of ∼50:1, spotted onto LB agar, and incubated at 37°C. As added points of reference, the E. coli strains containing the immunity proteins 14240 and 14260 were included in both of the experiments depicted in the left and right panels. After 2 h (A) or 24 h (B) of incubation, the numbers of E. coli CFU were determined by plating dilutions of the entire bacterial growth area on selective medium. In the 2-h incubation, the numbers of CFU of the vector control decreased, indicative of killing, whereas in the 24-h incubation, the numbers of CFU for the control increased somewhat, indicative of the combined effect of growth and killing (Fig. S2A). Thus, the percent changes in CFU for the vector control were set to 1, and others were normalized to that control (dashed horizontal line), in order to make clearer which cloned immunity proteins afforded protection against killing by S. maltophilia T4SS. Asterisks indicate those cases where there were significant differences in CFU obtained for the recombinant clone versus the control (*, *P* < 0.05; **, *P* < 0.01; ***, *P* < 0.001). Data are presented as the means and standard deviations of results from three independent experiments (*n* = 3 each).

**FIG 3:**
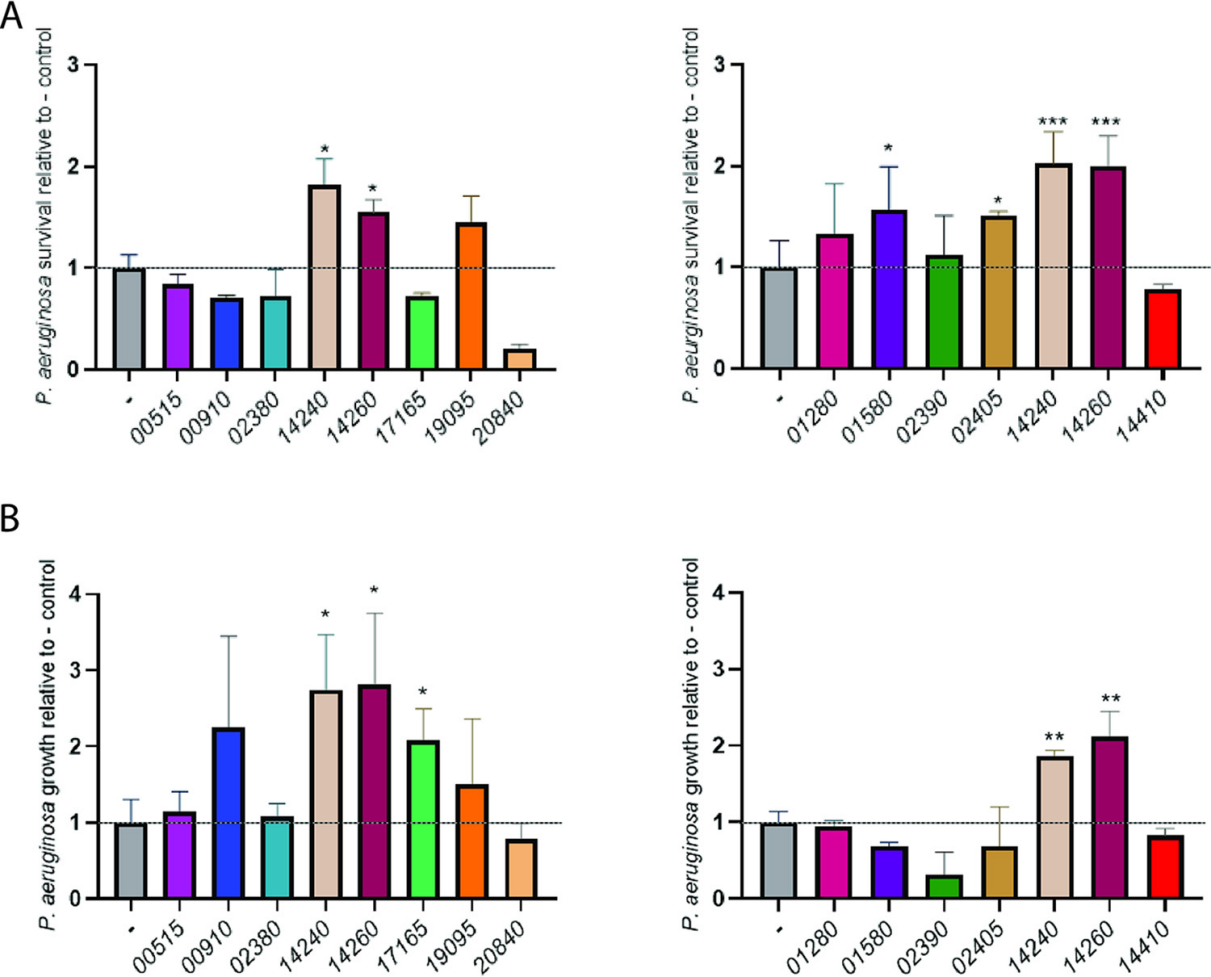
Ability of cloned immunity proteins to protect P. aeruginosa from killing by wild-type S. maltophilia. P. aeruginosa strain 7700 containing either the vector pBBR1MCS alone (-) or the cloned gene encoding one of the predicted immunity proteins from S. maltophilia K279a, i.e., 00515, 00910, 02380, 14240, 14260, 17165, 19095, or 20840 (left panels) or 01280, 01580, 02390, 02405, 14240, 14260, or 14410 (right panels), was mixed with S. maltophilia K279a in a (S. maltophilia/P. aeruginosa) ratio of ∼50:1, spotted onto LB agar, and incubated at 37°C. As added points of reference, E. coli strains containing the immunity proteins 14240 and 14260 were included in both of the experiments depicted in the left and right panels. After 2 h (A) or 24 h (B) of incubation, the numbers of P. aeruginosa CFU were determined by plating dilutions of the entire bacterial growth area on selective medium. In the 2-h incubation period, the numbers of CFU of the vector control decreased, indicative of killing, whereas during the 24-h incubation, the numbers of CFU for the control increased, indicative of the combined effect of growth and killing (Fig. S2B). Thus, the percent changes in CFU for the vector control were set to 1, and the others were normalized to that control (dashed horizontal line), in order to make clearer which cloned immunity proteins afforded protection against killing by S. maltophilia T4SS. Asterisks indicate those cases where there were significant differences in CFU obtained for the recombinant clone versus the control (*, *P* < 0.05; **, *P* < 0.01; ***, *P* < 0.001). Data are presented as the means and standard deviations of results from three independent experiments (*n* = 3 each).

To focus our mutagenesis efforts, we sought to identify which putative effectors had an immunity protein that conferred protection against the T4SS’s bactericidal activity. Immunity proteins normally provide self-protection to the organism encoding the T4SS (73, 74), but, for our purposes, we examined the effect of their expression in heterologous bacteria. Each of the 13 putative immunity proteins, determined based upon their arrangement in effector gene operons as described above, was cloned on a plasmid and introduced into Escherichia coli DH5α and P. aeruginosa 7700, the strains that we had previously shown are sensitive to antagonism by S. maltophilia K279a (31). Whereas DH5α is a laboratory strain of E. coli, 7700 is wild-type P. aeruginosa isolated from water. To begin, target E. coli bacteria containing or not containing the different immunity proteins were coincubated with T4SS-expressing K279a for 2 h at 37°C, and then their remaining numbers were determined. As previously found (31), there is normally a decrease in E. coli CFU during this short incubation period. Thus, any increase in the recovery of the heterologous bacteria (above the control) is evidence of protection against the T4SS. Six of the cloned immunity proteins, i.e., 14240, 14260, 17165, 20840, 01280, and 01580, allowed DH5α to survive better in the presence of K279a (Fig. 2A and Fig. S2A). When we extended the incubation to 24 h, 14240, 14260, 17165, 20840, and 01580 continued to give a protective effect, as one might expect (Fig. 2B and Fig. S2A). Another protein, 14410, showed a protective effect in the 24-h, but not 2-h, incubation (Fig. 2B). More significantly, when we incubated wild-type P. aeruginosa containing or not containing the different immunity proteins with strain K279a for 2 h at 37°C, four of the cloned proteins, i.e., 14240, 14260, 01580, and 02405, allowed the pseudomonad to survive better amid S. maltophilia (Fig. 3A and Fig. S2B). Among these four, 14240 and 14260 appeared to provide the greatest level of protection. Also, 14260 and 14240, but none of the others, showed clear evidence of protection when the coincubation was extended to 24 h (Fig. 3B and Fig. S2B). The immunity protein 17165 showed a protective effect in this 24-h incubation, although it had not done so in the 2-h incubation (Fig. 3B). That the putative immunity proteins 00515, 00910, 02380, 19095, and 02390 did not appear to provide any protection means either that they are not true immunity proteins or that they were not adequately expressed and/or localized in the E. coli or P. aeruginosa cells. Nonetheless, 14240 and 14260 stood out as clearly being important, since they (i) provided the greatest protection against the P. aeruginosa strain, in terms of both the magnitude of the protection and that it occurred at multiple time points (Fig. 3), and (ii) also blocked killing of a second bacterium (Fig. 2).

10.1128/mBio.01502-21.2 FIG S2 Raw data for Fig. 2 and 3, concerning the ability of cloned immunity proteins to protect E. coli and P. aeruginosa from killing by wild-type S. maltophilia. E. coli DH5α (A) and P. aeruginosa 7700 (B) containing either the vector pBBR1MCS alone (-) or a cloned gene encoding one of the indicated immunity proteins from S. maltophilia K279a were mixed with strain K279a in a (S. maltophilia/other bacterium) ratio of ∼50:1, spotted onto LB agar, and incubated at 37°C. As added points of reference, the E. coli or P. aeruginosa strain containing the immunity proteins 14240 and 14260 was included in both of the experiments depicted in the left and right panels. After 2 h (top row in panels A and B) or 24 h (bottom row in panels A and B) of incubation, the numbers of E. coli or P. aeruginosa CFU were determined by plating dilutions of the entire bacterial growth area on selective medium. The results presented are the numbers of CFU at *t* = 0 h and *t* = 2 h or *t* = 24 h, and the data are presented as the means and standard deviations of results from three independent experiments (*n* = 3 each). Download FIG S2, TIF file, 0.7 MB. Copyright © 2021 Nas et al. 2021 Nas et al. https://creativecommons.org/licenses/by/4.0/ This content is distributed under the terms of the Creative Commons Attribution 4.0 International license.

### Putative T4SS effectors 14245 and 14255 are required for the ability of S. maltophilia to compete against P. aeruginosa and E. coli.

**FIG 4:**
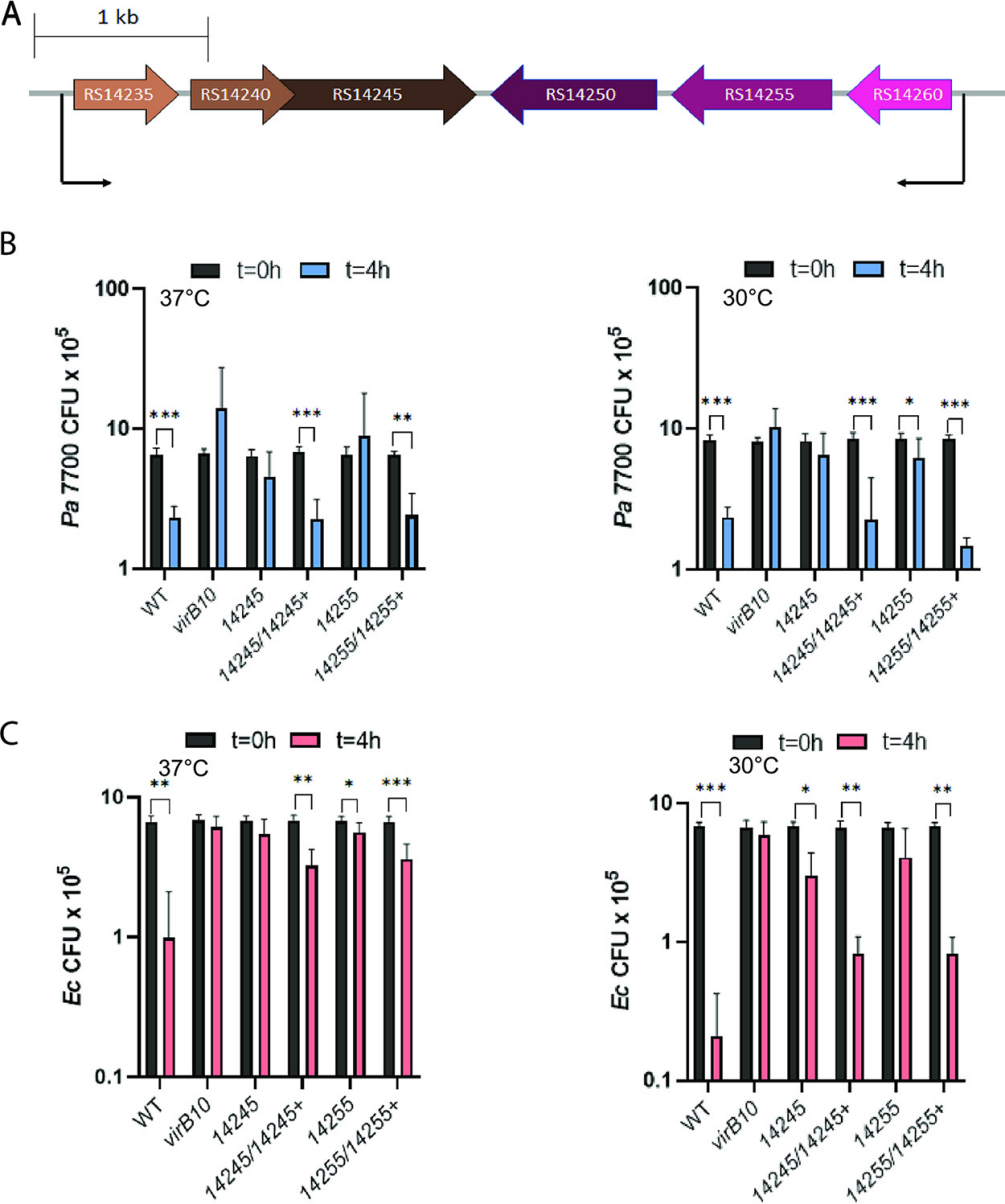
Bactericidal effect of S. maltophilia wild-type, *virB10* mutant, *14245* mutant, and *14255* mutant strains on P. aeruginosa *and* E. coli. (A) Representation of the region of the S. maltophilia K279a chromosome containing the operon with ORFs encoding effector 14245 and its cognate immunity protein (brownish horizontal arrows) and the neighboring operon encoding effector 14255 and its corresponding immunity protein 14260 (pink-purple horizontal arrows). Horizontal arrowheads indicate the direction of transcription, with the thin, bent arrows showing the approximate position of the predicted promoters. A scale, in kilobases, is presented in the upper left. (B and C) S. maltophilia (Sm) K279a (WT), *virB10* mutant NUS15 (*virB10*), 14245 mutant NUS17 (*14245*), complemented mutant NUS17 (pB*14245*) (*14245/14245*+), 14255 mutant NUS19 (*14255*), and complemented mutant NUS19 (pB*14255*) (*14255/14255*+) were mixed with P. aeruginosa strain 7700 (B) and E. coli strain DH5α (C) in a (S. maltophilia/other bacterium) ratio of ∼50:1 and spotted onto LB agar, and after 4 h of incubation at either 37°C (left panels) or 30°C (right panels), the numbers of each strain were determined by plating dilutions of the entire bacterial growth area on selective medium. The results presented are the numbers of P. aeruginosa CFU or E. coli CFU at *t* = 0 h and *t* = 4 h, with asterisks indicating those cases where there were significant differences in CFU obtained at *t* = 0 versus *t* = 4 h (*, *P* < 0.05; **, *P* < 0.01; ***, *P* < 0.001). Data are presented as the means and standard deviations of results from three independent experiments (*n* = 3 each).

The analysis of the immunity proteins implicated 14245 and 14255, i.e., the corresponding effectors of 14240 and 14260 (Fig. 1A), as the most likely mediators of the (most significant) bactericidal effect of the S. maltophilia T4SS. These putative effectors were encoded by all 42 sequenced strains of S. maltophilia that encode a T4SS and were highly conserved in amino acid sequences across the strains (Fig. 1A and Table S1A). Also, they were encoded by all sequenced *Stenotrophomonas* species that carry the T4SS (Fig. 1B). Interestingly, the operon encoding 14245 maps adjacent to but in the opposite direction from the operon encoding 14255 (Fig. 4A). Both of these operons had a third ORF, which encoded a hypothetical protein (Fig. 4A). Significantly, this locus maps very close and indeed is closer than any of the other putative effector genes to the locus encoding the T4SS apparatus, i.e., SMLT_RS14275 to SMLT_RS14330 (31). Proximity to the locus encoding the secretion apparatus is often a hallmark of secreted substrates (63, 75, 76).

To determine whether 14245 and 1455 have required roles in the antibacterial activity of the S. maltophilia T4SS, we constructed mutants of strain K279a that specifically lack either 14245 or 14255 and then tested them for their relative ability to kill P. aeruginosa 7700. As before (31), coincubation for a few hours at 37°C with K279a, but not its *virB10* T4SS mutant, led to an ∼3-fold reduction in the numbers of P. aeruginosa (Fig. 4B, left). When we did the coincubation at 30°C, as an initial look into how interactions might occur under environmental conditions, the effect of the T4SS was still present (Fig. 4B, right). As predicted by our analysis of the immunity proteins, the 14245 mutant had a diminished ability to impede the survival of P. aeruginosa, and this was evident at 37°C and 30°C (Fig. 4B). Reintroduction of the 14245 gene into the mutant restored its capacity to kill strain 7700, confirming that the 14245 protein is required for the ability of S. maltophilia to kill a heterologous bacterium. The 14255 mutant was also impaired for the ability to blunt the survival of P. aeruginosa, although the mutant phenotype was slightly more evident at 37°C than at 30°C (Fig. 4B). At both temperatures, a complemented 14255 mutant had restored activity, confirming that the 14255 protein, like the 14245 protein, is needed for the ability of S. maltophilia to maximally counteract its competitor. As we had observed previously for the wild type (WT) and the *virB10* mutant (31), the numbers of 14245 mutant and 14255 mutant did not change during the incubation period (Fig. S3A), indicating that the effects on P. aeruginosa depicted in Fig. 4B were not simply or indirectly due to alterations in growth of the S. maltophilia strains. The complemented mutants did show a drop in CFU during the coincubation at 37°C (but not at 30°C), which we suspect is due to elevated levels of the plasmid-encoded effectors not being sufficiently blocked by chromosomally encoded immunity proteins. Nonetheless, as noted above, the complemented mutants behaved as the wild type did in terms of impeding the survival of strain 7700, affirming that 14245 and 14255 are necessary for S. maltophilia to fully kill P. aeruginosa. The killing effects of 14245 and 14255 were evident whether the coincubations had a starting ratio of S. maltophilia to P. aeruginosa equal to 50 (as in Fig. 4B) or equal to 10 or 1 (Fig. S4). When we did the competition assay using strain DH5α, we confirmed the inhibitory effect of the T4SS on E. coli at 37°C (31) and further observed that the inhibition of the E. coli strain, like the inhibition of P. aeruginosa 7700, was present and somewhat greater at 30°C (Fig. 4C). Most importantly, the 14245 mutant and 14255 mutant were again impaired for the ability to kill the competitor, whereas their complements were better at countering E. coli, especially at 30°C (Fig. 4C). When we monitored the numbers of S. maltophilia strains during these coincubations, the wild type and the mutants displayed no changes in CFU but the complemented mutants showed some reduction (Fig. S3A), as we had observed for the incubations with P. aeruginosa. Thus, these data confirmed that 14245 and 14255 are necessary for the ability of S. maltophilia to optimally kill two types of heterologous bacteria. In some cases, e.g., during the 30°C incubation with P. aeruginosa 7700 or the 37°C incubation with E. coli DH5α, the 14245 mutant appeared to be as impaired as the *virB10* mutant was (Fig. 4B and C). In other cases, e.g., during 37°C incubation with strain 7700, the behavior of the 14255 mutant most closely matched the behavior of the *virB10* mutant (Fig. 4C). Thus, it appears that 14245 and 14255 are (the) major mediators of the bactericidal effects of the S. maltophilia T4SS, at least against the species tested here.

10.1128/mBio.01502-21.3 FIG S3 Numbers of S. maltophilia wild-type and mutant strains after coincubation with P. aeruginosa and E. coli. (A) S. maltophilia (*Sm*) K279a (WT), *virB10* mutant NUS15 (*virB10*), 14245 mutant NUS17 (*14245*), complemented mutant NUS17 (pB*14245*) (*14245/14245+*), 14255 mutant NUS19 (*14255*), and complemented mutant NUS19 (pB*14255*) (*14255/14255+*) were mixed with P. aeruginosa strain 7700 (top row) and E. coli strain DH5α (bottom row) in a (S. maltophilia/other bacterium) ratio of ∼50:1 and spotted onto LB agar, and after 4 h of incubation at either 37°C (left panels) or 30°C (right panels), the numbers of S. maltophilia strain K279a were determined by plating dilutions of the entire bacterial growth area on selective medium. (B) S. maltophilia K279a (WT), *virB10* mutant NUS15 (*virB10*), *02375* mutant NUS21 (*02375*), *19100* mutant NUS23 (*19100*), and *20845* mutant NUS24 (*20845*) were mixed with E. coli strain DH5α (top row) and P. aeruginosa strain 7700 (bottom row) in a (S. maltophilia/other bacterium) ratio of ∼50:1 and spotted onto LB agar, and after 4 h of incubation at either 37°C (left panels) or 30°C (right panels), the numbers of S. maltophilia strain K279a were determined by plating dilutions of the entire bacterial growth area on selective medium. The results presented are the numbers of CFU at *t* = 0 h and *t* = 4 h, with asterisks indicating those cases where there were significant differences in CFU obtained at *t* = 0 versus *t* = 4 h (*, *P* < 0.05; **, *P* < 0.01). Data are presented as the means and standard deviations of results from three independent experiments (*n* = 3 each). Download FIG S3, TIF file, 0.6 MB. Copyright © 2021 Nas et al. 2021 Nas et al. https://creativecommons.org/licenses/by/4.0/ This content is distributed under the terms of the Creative Commons Attribution 4.0 International license.

10.1128/mBio.01502-21.4 FIG S4 Bactericidal effect of S. maltophilia wild-type, *virB10* mutant, *14245* mutant, and *14255* mutant strains on P. aeruginosa 7700 when coincubated at different start ratios. S. maltophilia K279a (WT), *virB10* mutant NUS15 (*virB10*), 14245 mutant NUS17 (*14245*), and 14255 mutant NUS19 (*14255*) were mixed with P. aeruginosa (Pa) strain 7700 in a (S. maltophilia/P. aeruginosa) ratio of ∼10:1 (A) or 1:1 (B) and spotted onto LB agar, and after 4 h of incubation at either 37°C (left side of each panel) or 30°C (right side of each panel), the numbers of P. aeruginosa strain 7700 were determined by plating dilutions of the entire bacterial growth area on selective medium. The results presented are the numbers of P. aeruginosa CFU at *t* = 0 h and *t* = 4 h, with asterisks indicating those cases where there were significant differences in CFU obtained at *t* = 0 versus *t* = 4 h (*, *P* < 0.05). Data are presented as the means and standard deviations of results from three independent experiments (*n* = 3 each). Download FIG S4, TIF file, 0.3 MB. Copyright © 2021 Nas et al. 2021 Nas et al. https://creativecommons.org/licenses/by/4.0/ This content is distributed under the terms of the Creative Commons Attribution 4.0 International license.

**FIG 5:**
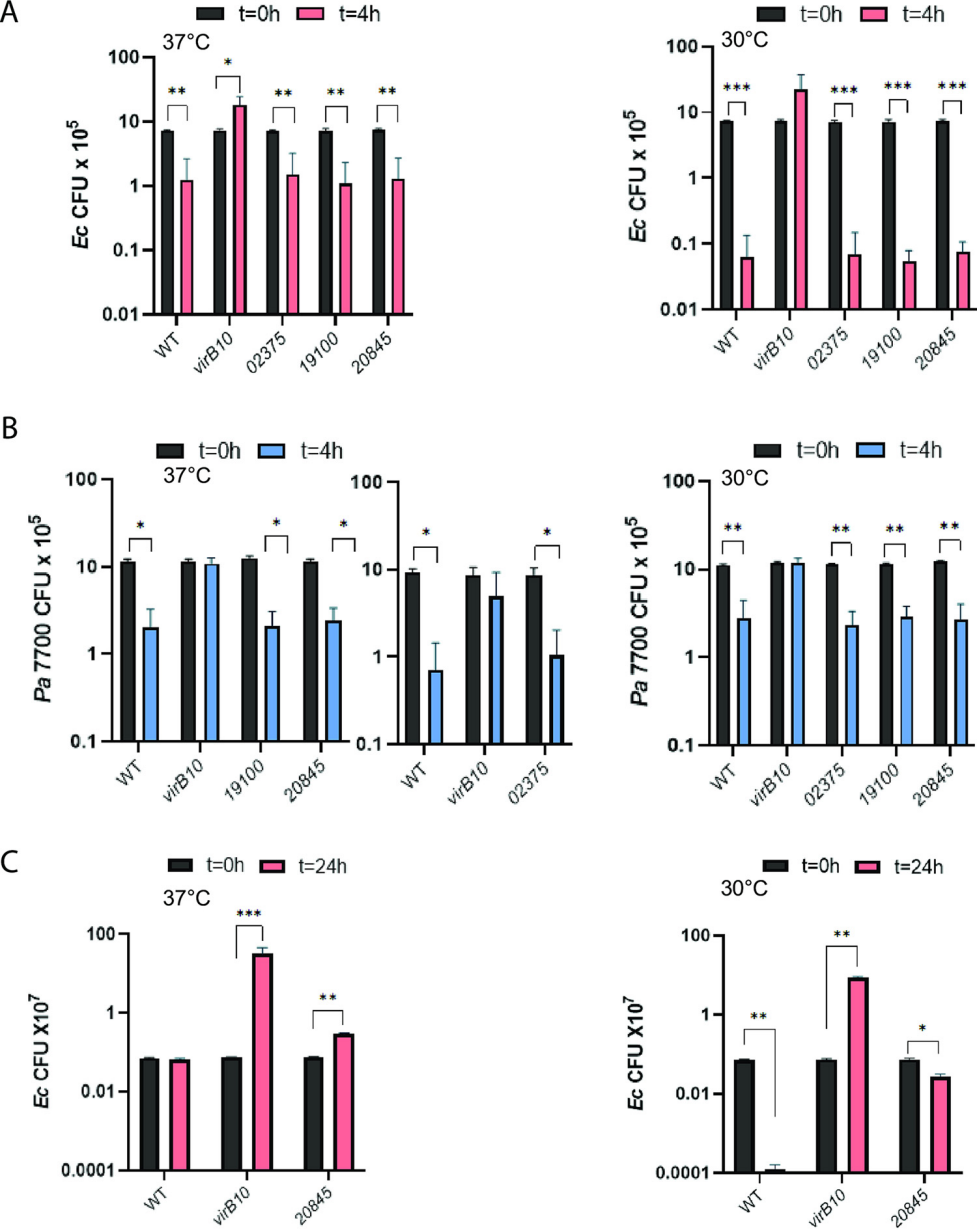
Effects of S. maltophilia wild-type, *virB10* mutant, *02375* mutant, *19100* mutant, and *20845* mutant strains on E. coli and P. aeruginosa. (A and B) S. maltophilia (Sm) K279a (WT), *virB10* mutant NUS15 (*virB10*), *02375* mutant NUS21 (*02375*), *19100* mutant NUS23 (*19100*), and *20845* mutant NUS24 (*20845*) were mixed with E. coli strain DH5α (A) and P. aeruginosa strain 7700 (B) in a (S. maltophilia/other bacterium) ratio of ∼50:1 and spotted onto LB agar, and after 4 h of incubation at either 37°C (left panels) or 30°C (right panels), the numbers of each strain were determined by plating dilutions of the entire bacterial growth area on selective medium. (C) S. maltophilia K279a WT, *virB10* mutant NUS15, and *20845* mutant NUS23 were mixed with E. coli DH5α at a ratio of ∼50:1 and spotted onto LB agar, and after 24 h of incubation at either 37°C (left panel) or 30°C (right panel), the numbers of CFU for each strain were determined. Results presented are the numbers of E. coli CFU or P. aeruginosa CFU at *t* = 0 h, *t* = 4 h, or *t* = 24 h, with asterisks indicating instances where there were significant differences in CFU obtained at *t* = 0 versus *t* = 4 or 24 h (*, *P* < 0.05; **, *P* < 0.01; ***, *P* < 0.001). Data are presented as the means and standard deviations of results from three independent experiments (*n* = 3 each).

To begin to discern if the other putative T4SS effectors have antibacterial activity, we constructed and tested three more mutants. We examined 20845, since it was annotated as a nuclease and is prevalent among S. maltophilia strains and *Stenotrophomonas* species (Fig. 1) and its immunity protein (20840) had partly protected E. coli from killing (Fig. 2A). We also tested 19100, since it was annotated as a lipase and is also well conserved (Fig. 1). Finally, we included 02375, as an example of a novel protein that is also well conserved (Fig. 1). Although each of the three newly made mutants survived just as well as the wild type did when coincubated with either P. aeruginosa 7700 or E. coli DH5α at 30°C and 37°C (Fig. S3B), none of them had an impaired ability to kill the heterologous bacteria upon short-term incubation (Fig. 5A and B). Since the immunity protein of 20845 had also afforded protection to E. coli during a 24-h coincubation with S. maltophilia (Fig. 2B), we further analyzed its corresponding mutant by extending the E. coli coincubation to 24 h. In this case, the 20845 mutant did show an impaired ability to compete against E. coli, and this was evident at both 37°C and 30°C (Fig. 5C). These data suggest that 20845 might also confer an antibacterial effect, although the effect of 20845 would appear to be bacteriostatic and more limited in terms of targets compared to 14245 and 14255. Thus, we returned our focus to the examination of 14245 and 14255, which had potent killing activities and were active against both E. coli and P. aeruginosa.

### 14245 and 14255 are bona fide substrates of the S. maltophilia T4SS.

**FIG 6:**
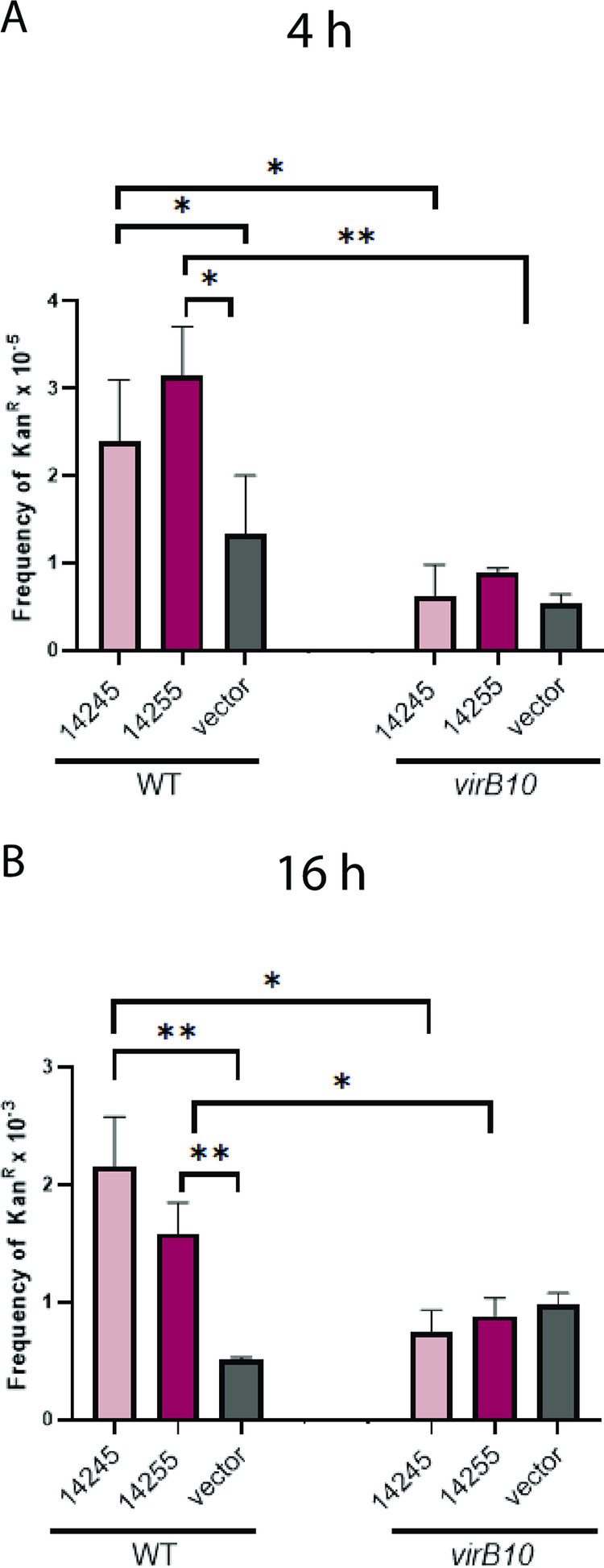
Translocation of effectors 14245 and 14255 from S. maltophilia wild-type and *virB10* mutant strains to heterologous bacteria. S. maltophilia K279a (WT) and *virB10* mutant NUS15 (*virB10*) either expressing a fusion between protein 14245 and the Cre recombinase (14245) or protein 14255 and the Cre recombinase (14255) or carrying only the pZL180Cre control (vector) were mixed with recipient E. coli XL1-Blue carrying pZL184 at a ratio of 1:50 (S. maltophilia/E. coli) and plated onto LB agar. After incubating at 37°C for 4 h (A) and 16 h (B), the entire spot of bacterial growth was resuspended and plated onto selective medium in order to identify excisants in which there was Cre-mediated excision of a floxed DNA sequence giving kanamycin resistance (Kan^r^). The transfer frequency was represented by the frequency of Kan^r^ CFU per donor bacterium. Indicative of effector protein translocation, asterisks indicate transfer frequencies from WT strains that were greater than that of either the vector control or those from the *virB10* mutant (*, *P* < 0.05; **, *P* < 0.01). Data are presented as the means and standard deviations of results from three independent experiments (*n* = 3 each).

**FIG 7:**
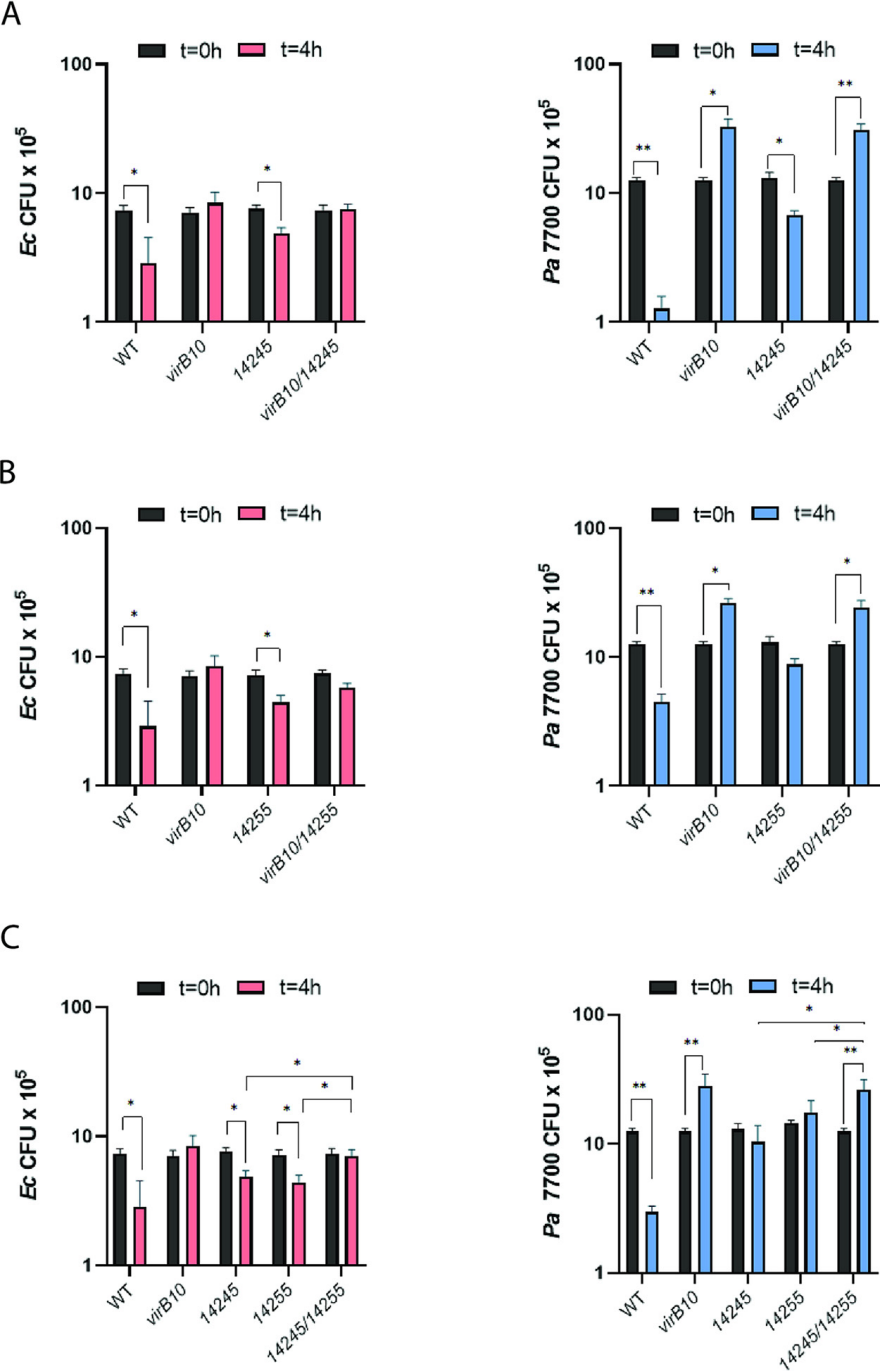
Bactericidal effect of S. maltophilia *virB10 14245* mutant, *virB10 14255* mutant, and *14245 14255* mutant strains on E. coli and P. aeruginosa. S. maltophilia K279a (WT) and *virB10* mutant NUS15 (*virB10*) (A to C) as well as either the *14245* mutant NUS17 (*14245*) and *virB10 14245* mutant NUS25 (*virB10 14245*) (A), *14255* mutant NUS19 (*14255*) and *virB10 14255* mutant NUS26 (*virB10 14255*) (B), or *14245* mutant, *14255* mutant, and *14245 14255* mutant NUS27 (*14245 14255*) (C) were mixed with E. coli DH5α (left panels) and P. aeruginosa strain 7700 (right panels) in a (S. maltophilia/other bacterium) ratio of ∼50:1 and spotted onto LB agar, and after 4 h of incubation at 37°C, the numbers of each strain were determined by plating dilutions of the entire bacterial growth area on selective medium. Results presented are the numbers of E. coli or P. aeruginosa CFU at *t* = 0 h and *t* = 4 h, with asterisks indicating cases where there were significant differences in CFU obtained at the two time points (*, *P* < 0.05; **, *P* < 0.01). Data are presented as the means and standard deviations of results from three independent experiments (*n* = 3 each).

To determine if 14245 and 14255 translocate into competing bacteria in a T4SS-dependent manner, we performed a Cre recombinase-mediated interbacterial protein translocation assay that has been used to study other T4SSs (66, 77). We generated translational fusions between the S. maltophilia effectors and the Cre recombinase in transfer-deficient plasmids and then introduced the constructs, along with the separate introduction of the vector control, into wild-type K279a and its *virB10* mutant. The different S. maltophilia strains were incubated with an E. coli strain that harbors a plasmid containing a *loxP*-flanked DNA segment which prevents the expression of a kanamycin resistance (Kan^r^) gene; i.e., Kan^r^ would be expressed only if, upon coincubation with S. maltophilia, the effector translocates Cre into the recipient leading to Cre-mediated excision of the floxed sequence. When we incubated the S. maltophilia donors with the E. coli recipient for 4 h at 37°C, the frequency of Kan^r^ resulting from incubation with wild-type K279a carrying either the 14245 or 14255 fusion was greater than that obtained upon incubation with wild-type S. maltophilia carrying the vector control that lacks an effector-Cre fusion (Fig. 6A), indicating that 14245 and 14255 are capable of translocating into a heterologous bacterium. Importantly, this translocation event was abolished when the donor was the *virB10* T4SS mutant (Fig. 6A). The same observations were made when we allowed the donor and recipient to incubate for 16 h (Fig. 6B). This indicated that 14245 and 14255 are translocated in a T4SS-dependent manner from S. maltophilia to a target bacterium. In order to gain further support for this conclusion, we constructed a double mutant inactivated for both *virB10* and *14245* and a double mutant lacking both *virB10* and *14255* and then tested the double mutants for their relative ability to kill E. coli and P. aeruginosa. We first observed that the *virB10 14245* mutant was not any more defective than the *virB10* mutant was (Fig. 7A), indicating that VirB10 (i.e., the T4SS apparatus) and 14245 operate in the same functional pathway. That the double mutant was more impaired than the 14245 mutant was compatible with there being more antibacterial effectors, such as 14255. Indeed, in the next experiment, we saw that the *virB10 14255* double mutant was not any more defective than the *virB10* mutant was (Fig. 7B), indicating that VirB10 and 14255 are functioning in the same pathway. These data, together with the results of the Cre recombinase assay, document that 14245 and 14255 are bona fide effectors of the S. maltophilia T4SS. With this confirmation, we sought to further investigate the degree to which 14245 and 14255 explain the bactericidal effect of the T4SS, as measured against P. aeruginosa 7700. Thus, we made and tested a double mutant lacking both *14245* and *14255*. This mutant was more defective for killing E. coli and P. aeruginosa 7700 than the 14245 mutant and 14255 mutant were (Fig. 7C), indicating that the two effectors promote independent killing mechanisms. However, the *14245 14255* mutant displayed a defect for killing similar to the *virB10* mutant (Fig. 7C), suggesting that 14245 and 14255 are required for all or nearly all T4SS-dependent killing that is occurring in this assay.

### S. maltophilia T4SS effector 14245 is sufficient to kill heterologous bacteria.

**FIG 8:**
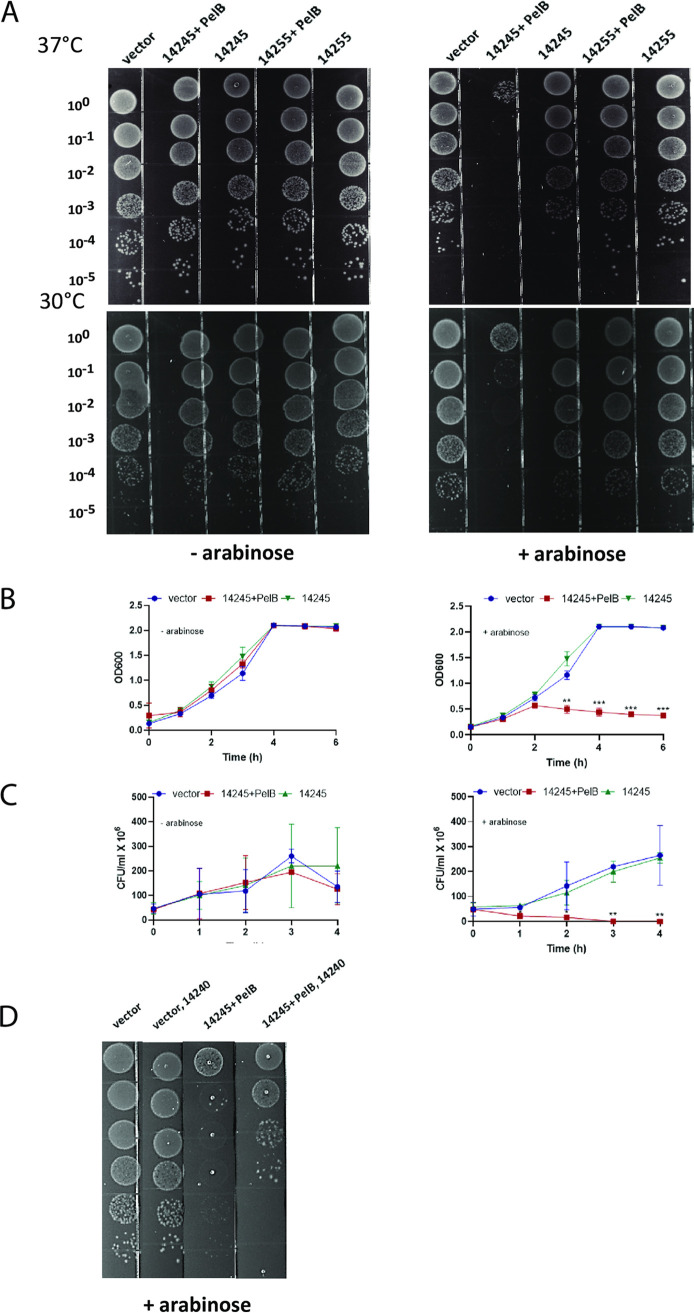
Effects of cloned T4SS effectors 14245 and 14255 on the viability of heterologous bacteria. (A) E. coli DH5α containing either the pBAD18 vector (vector), pBAD18-pelB14245 expressing 14245 with a PelB periplasmic-localization signal (14245 + PelB), pBAD18-14245 expressing 14245 without a periplasmic-localization signal (14245), pBAD18-pelB14255 expressing 14255 with a PelB periplasmic-localization signal (14255 + PelB), or pBAD18-14255 expressing 14255 without a periplasmic-localization signal (14255) was serially diluted (as indicated) and spotted onto LB agar without (left panel) and with (right panel) 0.4% arabinose, which induces expression of the cloned proteins from the vector’s pBRA promoter. Images present bacterial survival and growth after 18 h of incubation at 37°C (top row) or 30°C (bottom row) and are representative of the results seen in three independent experiments. (B and C) The E. coli strains indicated above in panel A were incubated at 37°C in LB broth without (left panels) and with (right panels) arabinose, and then at the indicated time points, bacterial survival and growth were assessed by measuring the OD_600_ of the cultures (B) and by determining the numbers of CFU by plating (C). Asterisks indicate time points at which the clone expressing periplasmically localized 14245 behaved differently from clones expressing the vector control or the cytoplasmically localized 14245 (*, *P* < 0.05; **, *P* < 0.01; ***, *P* < 0.001). (D) E. coli DH5α containing either pBAD18 (vector), pBAD18-pelB14245 (14245 + PelB), pBAD18 and a second vector expressing the 14240 immunity protein (vector, 14240), or pBAD18-pelB14245 and the immunity protein-encoding plasmid (14245 + PelB, 14240) was serially diluted and spotted as indicated in panel A. Clones with the 14240 construct were plated on LB plates that also contained kanamycin. Data are presented as the means and standard deviations of results from three independent experiments (*n* = 3 each).

Given the validation of the importance of 14245 and 14255, we sought to determine if expression of these effectors alone within another bacterium would be sufficient to affect viability or growth of that competitor. Given data from T6SS studies (74, 78) as well as the putative enzyme activities of 14245 and 14255 (Fig. 1A), we posited that the effectors act in the target cell’s periplasm. Thus, following a method that is long established in the T6SS field (79–81), we joined full-length 14245 and 14255 to an N-terminal periplasmic localization signal derived from the periplasmic protein PelB and then placed the genes’ expression under the control of an arabinose-inducible (pBRA) promoter, all in the vector pBAD18. E. coli DH5α containing these constructs as well as versions of 14245 and 14255 lacking the PelB signal were incubated on LB agar with and without arabinose at 37°C and 30°C. Cloned 14255, with or without periplasmic localization, did not alter the survival of E. coli, whether grown with or without inducing arabinose and whether grown at 37°C or 30°C (Fig. 8A), suggesting that 14255, although necessary for optimal killing by the S. maltophilia T4SS (as determined by mutant analysis), may not be sufficient to kill on its own and that it might have an interacting partner. It is also possible that the presence of the PelB sequence may have altered the activity of the 14255 protein. In contrast, clones containing induced, periplasmic 14245 showed greatly impaired survival at 37°C and 30°C, with the efficiency of plating being ∼4 orders of magnitude less than that of control E. coli (Fig. 8A, right). This massive killing was not seen when there was no induction of gene expression; i.e., in the absence of arabinose (Fig. 8A, left). Moreover, it did not occur when the induced, cloned protein lacked a periplasmic localization signal (Fig. 8A, right). These data indicated that 14245, when delivered into the periplasm, is sufficient to kill E. coli. To bolster this conclusion, we tested the effect of the pBRA-PelB-14245 and pBRA-14245 constructs, along with the pBRA control, on E. coli survival in broth. When there was no induction of gene expression, the optical densities (OD) of the different E. coli cultures were similar, but when arabinose was present, the periplasmically localized 14245 resulted in a sharp drop in the OD (Fig. 8B). The impact of 14245 was also evident when we assayed for CFU in the presence of arabinose; whereas there was an ∼5-fold increase in CFU in 4 h for the strain containing the vector or the 14245 gene lacking the PelB signal, the PelB-14245 fusion resulted in an immediate and steady decline in CFU (Fig. 8C, right). Finally, we observed that the coexpression of the immunity protein 14240 reversed to a large extent the killing activity of the periplasmic PelB-14245 (Fig. 8D). In sum, these data indicate that the T4SS effector 14245 is both necessary and sufficient to kill heterologous bacteria.

### T4SS effectors 14245 and 14255 promote the ability of S. maltophilia to kill clinical isolates of P. aeruginosa.

**FIG 9:**
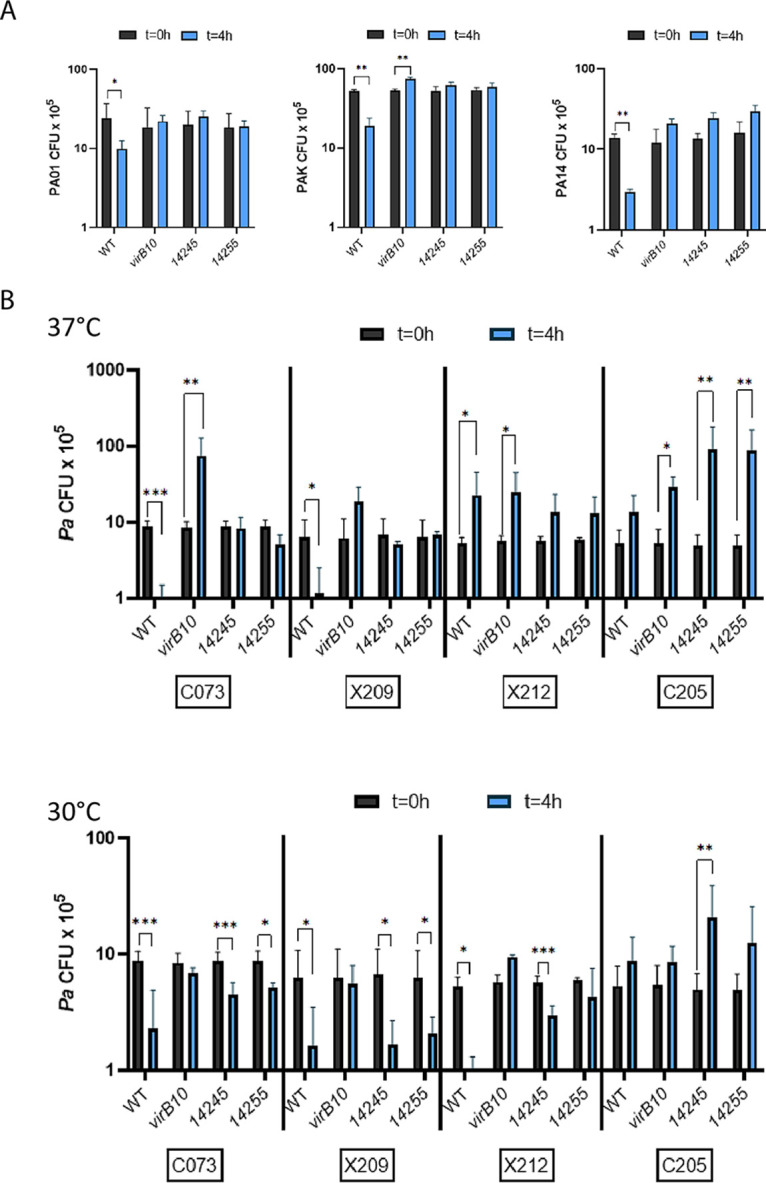
Bactericidal effect of S. maltophilia wild-type, *virB10* mutant, *14245* mutant, and *14255* mutant strains on clinical isolates of P. aeruginosa. (A) S. maltophilia K279a (WT), *virB10* mutant NUS15 (*virB10*), 14245 mutant NUS17 (*14245*), and 14255 mutant NUS19 (*14255*) were mixed with P. aeruginosa strains PAO1, PAK, and PA14 in a (S. maltophilia/other bacterium) ratio of ∼50:1 and spotted onto LB agar, and after 4 h of incubation at 37°C, the numbers of each strain were determined by plating dilutions of the entire bacterial growth area on selective medium. (B) The four S. maltophilia strains indicated in panel A were mixed with P. aeruginosa CF lung isolates C073, X209, X212, and C205 in a ratio of ∼50:1 and spotted onto LB agar, and after 4 h of incubation at 37°C (top) or 30°C (bottom), the numbers of each strain were determined by plating. The results presented are the numbers of P. aeruginosa CFU at *t* = 0 h and *t* = 4 h, with asterisks indicating those cases where there were significant differences in CFU obtained at *t* = 0 versus *t* = 4 h (*, *P* < 0.05; **, *P* < 0.01; ***, *P* < 0.001). Values that are not sufficiently clear from the figure are as follows: at 37°C, C073 CFU when competed against WT = 7.1 × 10^4^; at 30°C, X212 CFU when competed against WT = 5.8 × 10^4^. Data are presented as the means and standard deviations of results from three independent trials (*n* = 3 each).

With the confirmation that 14245 and 14255 are indeed T4SS substrates that help mediate the killing of and competition with an environmental isolate of P. aeruginosa, we sought to determine if the two have activity against clinical isolates of P. aeruginosa. Based upon testing the *virB10* mutant and its complement, we had previously reported that S. maltophilia T4SS promotes the killing of P. aeruginosa strain PAO1, a human wound isolate (82), and P. aeruginosa PAK, another well-studied clinical isolate (83, 84). Shortly thereafter (58), it was reported that the *Stenotrophomonas* T4SS also impedes P. aeruginosa PA14, a human burn wound isolate (85, 86). When our S. maltophilia strains were competed against these three P. aeruginosa strains in parallel, the *virB10* mutant again showed an impaired ability to kill each of the strains and to comparable degrees (Fig. 9A). More importantly to the present study, the 14245 mutant and 14255 mutant were each as impaired as the *virB10* mutant was against the three targets (Fig. 9A), further implicating 14245 and 14255 as having roles in the bactericidal effects of the S. maltophilia T4SS. As before, the numbers of mutant bacteria were constant during the incubation (Fig. S5A), indicating that the effects on P. aeruginosa shown in Fig. 9A were not simply due to alterations in growth of the S. maltophilia strains. Since PAO1, PAK, and PA14 were isolated more than 40 years ago and have been passaged a great deal in laboratories, we studied, at both 37°C and 30°C, the bactericidal effect of the S. maltophilia T4SS and effectors 14245 and 14255 against four strains of P. aeruginosa that were recently isolated from the lungs of four CF patients. Based upon mutant analysis, the T4SS promoted killing of two of these isolates, i.e., C073 and X209, and in both cases, the killing was greater at 37°C than at 30°C, and effectors 14245 and 14255 continued to play a major role, particularly at 37°C (Fig. 9B, two leftmost panels). When we monitored the numbers of the S. maltophilia strains during the coincubations, the wild type and the mutants behaved similarly, aside from a slight reduction for the 14245 mutant at 30°C (Fig. S5B), indicating that the more notable effects on P. aeruginosa depicted in Fig. 9B were not due to alterations in growth of the S. maltophilia strains. A third P. aeruginosa isolate from the CF lung, strain X212, was sensitive to killing by the S. maltophilia T4SS and the 14245 effector, but this was seen only at the lower temperature (Fig. 9B), and in the case of the effector, could not be simply ascribed to there being greater growth of the mutant (Fig. S5B). Further examination of complemented mutants confirmed the importance of the 14245 and 14255 effectors in the killing of these three clinical isolates (Fig. S6). In marked contrast, the S. maltophilia T4SS (and its effectors) did not promote the killing of the fourth isolate, strain C205, at either temperature (Fig. 9B), even though the S. maltophilia strains were largely able to maintain their numbers (Fig. S5B). Together, these data confirm that the S. maltophilia T4SS and effectors 14245 and 14255 are active against multiple clinical isolates of P. aeruginosa, including contemporary isolates obtained from the lungs of CF patients.

10.1128/mBio.01502-21.5 FIG S5 Numbers of S. maltophilia wild-type, *virB10* mutant, *14245* mutant, and *14255* mutant strains after coincubation with clinical isolates of P. aeruginosa. (A) S. maltophilia (*Sm*) K279a (WT), *virB10* mutant NUS15 (*virB10*), 14245 mutant NUS17 (*14245*), and 14255 mutant NUS19 (*14255*) were mixed with P. aeruginosa strains PAO1, PAK, and PA14 in a (S. maltophilia/P. aeruginosa) ratio of ∼50:1 and spotted onto LB agar, and after 4 h of incubation at 37°C, the numbers of strain K279a CFU were determined by plating dilutions of the entire bacterial growth area on selective medium. (B) The four S. maltophilia strains indicated in panel A were mixed with P. aeruginosa CF lung isolates C073, X209, X212, and C205 in a ratio of ∼50:1 and spotted onto LB agar, and after 4 h of incubation at 37°C (top) or 30°C (bottom), the numbers of K279a were determined by plating. The results presented are the numbers of CFU at *t* = 0 h and *t* = 4 h, with asterisks indicating those cases where there were significant differences in CFU obtained at *t* = 0 versus *t* = 4 h (*, *P* < 0.05; **, *P* < 0.01; ***, *P* < 0.001). Data are presented as the means and standard deviations of results from three independent experiments (*n* = 3 each). Download FIG S5, TIF file, 0.7 MB. Copyright © 2021 Nas et al. 2021 Nas et al. https://creativecommons.org/licenses/by/4.0/ This content is distributed under the terms of the Creative Commons Attribution 4.0 International license.

10.1128/mBio.01502-21.6 FIG S6 Bactericidal effect of S. maltophilia wild-type, *virB10* mutant, *14245* mutant, *14255* mutant, and complemented mutant strains on clinical isolates of P. aeruginosa. S. maltophilia K279a (WT), *virB10* mutant NUS15 (*virB10*), 14245 mutant NUS17 (*14245*), complemented mutant NUS17 (pB*14245*) (*14245/14245+*), 14255 mutant NUS19 (*14255*), and complemented mutant NUS19 (pB*14255*) (*14255/14255+*) were inoculated onto LB agar in a starting ratio of ∼50:1 with either P. aeruginosa (*Pa*) strains C073 and X209 at 37°C (A) or P. aeruginosa strain X212 at 30°C (B), and then after 4 h, the numbers of P. aeruginosa were determined by plating. The results presented are the numbers of CFU at *t* = 0 h and *t* = 4 h, with asterisks indicating those cases where there were significant differences in CFU obtained at *t* = 0 versus *t* = 4 h (*, *P* < 0.05; **, *P* < 0.01; ***, *P* < 0.001). Values that are not sufficiently clear from the figure are as follows: in panel A, C073 CFU when competed against WT = 7.0 × 10^4^, X209 CFU when competed against WT = 5.2 × 10^3^, and X209 CFU when competed against the complemented 14245 mutant = 9.8 × 10^4^, and in panel B, X212 CFU when competed against the WT = 4.9 × 10^4^. Data are presented as the means and standard deviations of results from three independent experiments (*n* = 3 each). Download FIG S6, TIF file, 0.3 MB. Copyright © 2021 Nas et al. 2021 Nas et al. https://creativecommons.org/licenses/by/4.0/ This content is distributed under the terms of the Creative Commons Attribution 4.0 International license.

## DISCUSSION

The present study represents the first documentation of a bactericidal effector of the recently described S. maltophilia T4SS and one of only a few such effectors from T4SSs overall. We designate the gene encoding 14245 as *tfcA*, for type four secreted bactericidal effector A, and hence the protein as TfcA. Furthermore, we designate the gene encoding 14255 as *tfcB*, for type four secreted bactericidal effector B, and hence the protein as TfcB. Discovering these roles for TfcA and TfcB further validates a bioinformatic screen that was developed for the study of *Xanthomonas* T4SS (65). While analyzing TfcA and TfcB, we also documented, for the first time, that the T4SS of S. maltophilia is active against recent clinical isolates, namely P. aeruginosa from the lungs of CF patients. The analysis of the bactericidal T4SS of S. maltophilia remains the only such examination of a human pathogen, since other experimental work on T4SS-mediated interbacterial competition involves *Xanthomonas* and *Bartonella* species that afflict plants and animals, respectively (65, 66). Yet, our findings have implications for not only the uncharacterized T4SSs that exist in other species of *Stenotrophomonas* but also T4SSs beyond *Stenotrophomonas* (31, 87).

The ∼46-kDa TfcA effector was both necessary and sufficient to kill heterologous bacteria. Based on the assays utilizing the PelB localization sequence, TfcA likely kills when it is injected by the T4SS into the periplasm of target bacteria. Compatible with this hypothesis is that the cognate immunity protein of TfcA carries a lipoprotein signal sequence, which would carry it to the periplasm of S. maltophilia where its protective effect is normally needed against “accidental leakage” of TfcA. Such a scenario is akin to that of many bactericidal effectors of T6SS (79–81). TfcA has very high sequence similarity to a large number of hypothetical proteins encoded by a wide range of bacteria, with the greatest levels of similarity occurring with proteins from the human pathogen Acinetobacter baumannii and environmental *Streptomyces* and *Xanthomonas* species (see Fig. S7A in the supplemental material). Importantly, the T4SS effector also has significant similarity to known lipases produced by five fungal species and three (Gram-negative) bacterial species (Fig. S7A), including members of the class III family of lipases and di- and triacylglycerol lipases (88–94). That TfcA might be a lipase is supported by structural prediction obtained using Phyre2 (Fig. S7B). Thus, we hypothesize that, following delivery by the T4SS, TfcA acts on the target’s lipid-containing, outer membrane and/or inner membrane, resulting in death of the competitor. Although ∼37-kDa TfcB was necessary for optimal killing by the S. maltophilia T4SS, it alone was not sufficient to kill targets whether expressed in the periplasm (due to a fused PelB sequence) or in the cytoplasm (when lacking the PelB tag). Thus, we posit that TfcB might have a T4SS-dependent, interacting partner that is needed for it to act. Alternately, it is possible that our assay system did not properly deliver (sufficient levels of) TfcB in order to see its toxic effect. Regardless of whether TfcB acts alone or in combination, we strongly suspect that its bactericidal activity is associated with a predicted lysozyme-like activity. TfcB has very high sequence similarity to a large number of hypothetical proteins that are encoded by a wide range of bacteria and are variously annotated as putative chitinases, putative lytic enzymes, and putative peptidoglycan-binding proteins (Fig. S8A). The greatest levels of similarity occurred with proteins from A. baumannii, P. aeruginosa, and environmental *Streptomyces*, *Mesorhizobium*, and *Xanthomonas* species. Yet, this T4SS effector also has significant similarity to at least two characterized enzymes, i.e., a T4SS-dependent and bactericidal peptidoglycan hydrolase from *X. citri* and a chitinase from Streptomyces griseus (Fig. S8A) (65, 95, 96). Compatible with these various BLAST results, Phyre2 software predicts a structure that fits into the family of endolysins (Fig. S8B). Thus, we posit that, following injection into the target cell, TfcB acts to degrade peptidoglycan, resulting in the death of the target cell. Compatible with this hypothesis is that the cognate immunity protein of TfcB is homologous to known peptidoglycan hydrolase inhibitors (65, 97). That S. maltophilia would utilize more than one effector to kill a single target, whether that is E. coli or P. aeruginosa, is compatible with the literature involving other antibacterial systems such as the T6SSs (73, 98). On the other hand, it is also conceivable that the importance of TfcA and TfcB and perhaps other putative effectors varies with the target. Future work should confirm how these newly defined, major effectors kill heterologous bacteria.

10.1128/mBio.01502-21.7 FIG S7 Protein homologs of and structural prediction for S. maltophilia T4SS effector 14245. (A) Using BLAST, proteins showing significant similarity to the effector 14245 were identified in GenBank at NCBI. Homologs are listed in the order of highest to lowest level of sequence identity, as indicated in the third column, with the corresponding E value in the final column. The species of origin for the homolog(s) are listed in the first column followed by the annotation for the protein in the second column. Whereas the first 20 entries represent uncharacterized “hypothetical” proteins, the final 8 are known enzymes whose lipase activity and/or structure has been defined. (B) Using Phyre2, a structural prediction places the 14245 effector within the class III family of lipases, as exemplified by a group of characterized fungal enzymes. Download FIG S7, TIF file, 0.7 MB. Copyright © 2021 Nas et al. 2021 Nas et al. https://creativecommons.org/licenses/by/4.0/ This content is distributed under the terms of the Creative Commons Attribution 4.0 International license.

10.1128/mBio.01502-21.8 FIG S8 Protein homologs of and structural prediction for T4SS effector 14255. (A) Using BLAST, proteins showing significant similarity to the effector 14255 were identified in GenBank at NCBI. Homologs are listed in the order of highest to lowest level of sequence identity, as indicated in the third column, with the corresponding E value in the final column. The species of origin for the homolog(s) are listed in the first column followed by the annotation for the protein in the second column. Whereas approximately 30 entries represent uncharacterized “hypothetical” proteins, 2 entries (which are denoted by the **) are known enzymes. (B) Using Phyre2, a structural prediction of the 14255 effector indicates that the protein is a member of the endolysin family. Download FIG S8, TIF file, 0.8 MB. Copyright © 2021 Nas et al. 2021 Nas et al. https://creativecommons.org/licenses/by/4.0/ This content is distributed under the terms of the Creative Commons Attribution 4.0 International license.

Aside from our extensive analysis of TfcA and TfcB, we gained evidence for there being antibacterial activities associated with other putative effectors of the S. maltophilia T4SS. The supporting data were greatest in the case of 20845, a putative nuclease that is well conserved among S. maltophilia strains and confers at least a bacteriostatic activity when delivered into some target bacteria. The putative effectors 01275, 01575, 02400, and 17170 might each also encode a bactericidal activity; however, mutant analysis obviously needs to be done in order to affirm the role of these proteins. That 17170 might prove to be important even though it lacks the typical C-terminal secretion signal of the other effectors indicates that there is utility to continuing to use multiple bioinformatic screens when studying S. maltophilia T4SS. The last other protein to mention as a bacteriostatic factor is the 14405 protein. This last case is supported by a recent study which determined that 14405 (i) is translocated from S. maltophilia into a laboratory strain of E. coli in a T4SS-dependent manner, (ii) is inhibitory to E. coli growth when expressed as a cloned protein within the periplasm, and (iii) is inhibitory to E. coli growth when delivered by the T4SS of *X. citri,* with this inhibitory effect being reversed by the presence of the cognate immunity protein (58). Thus, despite the clear role of TfcA and TfcA in the bactericidal activity of S. maltophilia, there are benefits to confirming if other proteins identified by bioinformatic analysis are bona fide substrates of the T4SS and, if they are, to characterizing their activities. The possibility that the S. maltophilia T4SS might elaborate as many as 13 (or perhaps more) effectors is compatible with the output of some of the other T4SSs (99, 100).

S. maltophilia and P. aeruginosa are important human pathogens that can coexist both in environmental niches, including water systems in hospital settings (101–108), and within the human host, most notoriously in the CF lung (3, 11, 109–113). Reports have varied as to whether each organism benefits from the other’s presence or not (11, 113). The VirB/D4 T4SS and the effectors TfcA and TfcB that we have defined obviously act to antagonize P. aeruginosa. For several reasons, we infer that this antagonism is biologically relevant. First, the T4SS and its effectors were active against a range of wild-type P. aeruginosa strains, including recent isolates from CF lungs. Second, the bactericidal effect was evident both at multiple temperatures, indicative of environmental habitats and the human host, and at bacterial ratios as low as 1:1. Third, based on our prior study (31), other strains of S. maltophilia can kill a P. aeruginosa competitor. Interestingly, one out of the four CF isolates tested (i.e., C205) was not impeded by S. maltophilia K279a. This could be due to that bacterium being resistant to the action of the T4SS and/or because it has its own antibacterial tools that weaken the *Stenotrophomonas* competitor (63, 114, 115). Given our results, it will be worthwhile to test an even larger panel of P. aeruginosa strains for sensitivity to S. maltophilia effectors. Moreover, it will be instructive to examine this impact of the T4SS under different assay conditions, such as in environmental samples and biofilms, on host cell surfaces, or in lungs of experimental animals. Although we featured in our studies the impact of the S. maltophilia T4SS on Pseudomonas species, the analysis is worth expanding to other species, especially those that naturally coexist with S. maltophilia in humans, whether it be in the CF lung, the SARS-CoV-2-infected lung, or other sites of mixed infection (14–16). Moreover, since we previously discovered that the S. maltophilia T4SS modulates death pathways in human cells (31), it will be important to ascertain if any of the effectors or putative effectors defined here (also) mediate interactions with mammalian targets. In conclusion, our results provide fresh insights into understanding the emerging pathogen S. maltophilia, the expanding role of T4SSs and their myriad effectors, and the principles of clinically relevant, interbacterial competition.

## MATERIALS AND METHODS

### Bacterial strains and media.

S. maltophilia K279a (American Type Culture Collection [ATCC] BAA-2423) was the primary wild-type strain of S. maltophilia used in this study as well as the parent for the *virB10* mutant NUS15 (31) and newly made mutants (see below). In addition to strain 7700 (ATCC 7700) (31), strains of P. aeruginosa used in the bacterial competition assays were PAO1, PAK, PA14, and CF lung isolates C073, C205, X209, and X212 (obtained from Alan Hauser, Northwestern University). In addition to also being employed in the competition assays, as before (31), E. coli DH5α served as the host for recombinant plasmids. E. coli S17-1 was used for conjugation during mutagenesis, as before (31), whereas E. coli XL1-Blue was used in the protein translocation assay, as previously described (77). All strains were maintained or tested at either 37°C or 30°C on Luria-Bertani (LB) agar (Becton, Dickinson). Wild-type and mutant S. maltophilia and recombinant DH5α were also grown and monitored in liquid cultures by using LB broth.

### DNA, RNA, and protein analysis.

S. maltophilia K279a DNA and RNA were isolated as before (21, 22). DNA sequence analysis and primer design were done using SnapGene (GSL Biotech). qRT-PCR analysis was performed as previously described (116), with the primers targeting putative T4SS effectors designed using the Primer-BLAST tool at NCBI. All primers (Integrated DNA Technology) are listed in Table S2 in the supplemental material. The T4SEpre and S4TE programs were utilized to search the K279a genome in GenBank at NCBI for putative T4SS effectors based on the presence of C-terminal sequence patterns (67, 68). Additionally, BLAST was used to search the K279a database for proteins bearing sequence similarity to GLxRIDHV and FAVQGxxDPAHxRAHV, the conserved C-terminal domains in *Xanthomonas* T4SS substrates (65, 71). The ORFs for the identified proteins were visualized on NCBI to determine whether the ORFs’ operons contained an associated immunity protein. Annotations of the putative effectors and immunity proteins were obtained using NCBI BLAST. A reciprocal best BLAST strategy and HHpred (117) were utilized to find orthologs of the S. maltophilia K279a T4SS effectors and immunity proteins within genomes deposited in GenBank. In that analysis, an E value cutoff of 1E−5 was used to remove highly divergent sequences. Structural homology and structural predictions for 14245 and 14255 were done by inputting the FASTA sequences for the proteins into the Phyre2 software (118).

10.1128/mBio.01502-21.10 TABLE S2 Primers used in this study. Download Table S2, PDF file, 0.1 MB. Copyright © 2021 Nas et al. 2021 Nas et al. https://creativecommons.org/licenses/by/4.0/ This content is distributed under the terms of the Creative Commons Attribution 4.0 International license.

### Mutant and complemented mutant constructions.

We introduced deletion mutations into the S. maltophilia K279a genes encoding 14245, 14255, 02375, 19100, and 20845, as we did before for various other genes (31). DNA fragments consisting of the FLP recombination target (FRT)-flanked chloramphenicol cassette bounded on the one side by the ∼600 bp of sequences upstream of the effector gene and flanked on the other side by the ∼600 bp of sequences downstream of the effector gene were synthesized by Twist Bioscience. Each of the constructs was ligated into pEX18Tc, and then the resultant plasmids were transformed into E. coli strain S17-1 followed by transfer to S. maltophilia K279a by conjugation. The desired gene deletions were confirmed by sequencing and PCR using target-gene specific primers (Table S2). Two independent 14245 mutants were obtained and were designated strains NUS17 and NUS18. Two independent 14255 mutants were made and were named strains NUS19 and NUS20. The two mutants inactivated for 02375 was designated strains NUS21 and NUS22. Finally, the 19100 mutant was named NUS23, and the 20845 mutant was named NUS24. For *trans*-complementation of the *14245* mutant NUS17 and the *14255* mutant NUS19, PCR fragments containing the *14245* and *14255* ORFs and their promoters were PCR amplified from K279a DNA using primer pairs MN58 and MN59 and MN60 and MN61, respectively (Table S2). These fragments were digested by EcoRI and XbaI and cloned into pBBR1MCS, yielding pB*14245* and pB*14255*, which were electroporated into their corresponding mutants. Transformants were selected on chloramphenicol-containing LB agar, and clones were confirmed as carrying pB*14245* and pB*14255* by PCR using M13 primers (Table S2). Double mutants lacking either *virB10* (i.e., SMLT_RS14310) and *14245* or *virB10* and *14255* were constructed by mobilizing pEXΔ*virB10*::*frt-cat-frt* carried by E. coli S17-1 (31) into either the newly made *14245* mutant NUS17 or *14255* mutant NUS19. The *virB10 14245* double mutant was designated strain NUS25, and the *virB10 14255* double mutant was designated NUS26. A double mutant lacking both 14245 and 14255 (designated NUS27) was constructed by mobilizing pEXΔ*14245*::*frt-cat-frt* carried by the E. coli S17-1 strain (above) into the NUS19 mutant containing the *14255* deletion.

### Assays for interbacterial killing and competition.

Aside from utilizing a range of incubation temperatures, interbacterial killing and competition were assessed as before (31). Briefly, strains to be tested were grown in LB broth at either 37°C or 30°C, and then the different cell suspensions brought to equivalent CFU/ml were combined to yield ratios of S. maltophilia to the heterologous bacterium approximating 50:1, 10:1, and 1:1. Fifty microliters of the mixture was spotted onto LB agar plates and incubated at 37°C or 30°C, either for 2 to 4 h in order to gauge bactericidal effects or for 24 h in order to judge effects on bacterial growth. Bacterial survival was determined by measuring the numbers of different CFU in the spot by plating resuspended material on LB agar. In order to test the complemented mutants in this assay and induce the expression of the plasmid-encoded S. maltophilia genes, 1 μM IPTG was present in both the LB broth used for the precultures and the LB agar. In order to measure the ability of strain K279a immunity proteins to protect heterologous bacteria against the S. maltophilia T4SS, the proteins’ coding regions were either synthesized by Twist Bioscience or PCR amplified by primers MN62 to MN71 (Table S2), digested by EcoRI and XbaI, and ligated into pBBR1MCS. The constructs were then transformed into E. coli DH5α and P. aeruginosa 7700, and the resultant transformants were incorporated into the bacterial killing and competition assays as described above.

### Cre recombinase-based assay for measuring bacterial translocation of T4SS effectors.

To measure the T4SS-dependent transfer or translocation of proteins from S. maltophilia strain K279a to a competing bacterium, we adapted a protein translocation assay developed to study the T4SS of Legionella pneumophila (77). DNA fragments containing either the ORF encoding 14245 or the ORF for 14255 were PCR amplified using primer pairs MN72 and MN73, and MN74 and MN75, respectively (Table S2), digested with BglII and KpnI, and then ligated to the 3′ end of *cre* on pZL180, which lacks an origin of transfer (*oriT*) (77). The resultant pZL180Cre*14245* and pZL180Cre*14255,* along with vector control pZL180Cre, were separately transformed into both wild-type K279a and its *virB10* mutant derivative. The newly made S. maltophilia donor strains and E. coli XL1-Blue carrying pZL184, a derivative of *mob*- pBBRMCS that carries a *loxP*-flanked KanR (77), were each grown to mid-log phase, washed twice with phosphate-buffered saline (PBS), and then resuspended to an OD_600_ of 0.3 in LB broth. The two cell suspensions were then combined, at a ratio of 1:50 (S. maltophilia/E. coli) in a volume of 1 ml. Fifty microliters of the mixtures was plated onto LB agar containing 1 μM IPTG and incubated at 37°C. After 4 h and 16 h of incubation, the entire spot was swabbed from the plate and resuspended in 1 ml of PBS. The desired excisants were then selected on LB agar containing 5% (wt/vol) sucrose, 30 μg/ml kanamycin, and 30 μg/ml chloramphenicol, i.e., a condition that kills both the donor S. maltophilia and E. coli recipients still carrying a floxed kanamycin cassette. The amount of donor S. maltophilia was determined by plating appropriate dilutions onto LB agar containing 100 μg/ml carbenicillin. Ultimately, the transfer frequency was represented by the frequency of kanamycin resistance CFU (i.e., the number of excisants) per donor bacterium, as previously defined (77).

### Assay for the bactericidal activity of cloned T4SS effectors.

In order to measure the bactericidal activity of S. maltophilia T4SS effectors, we utilized a method that was established in the T6SS field and also permits the identification of effectors that kill by acting within the periplasm (79–81). DNA fragments containing the full-length 14245 gene or the entire 14255 gene were PCR amplified using pairs MN58 and MN59 and MN60 and MN61, respectively (Table S2), digested with EcoRI and XbaI, and then ligated into pBAD18 (119) such that the expression of the cloned genes was under the control of the arabinose-inducible pBRA promoter. Once transformed into target E. coli, these constructs, designated pBAD18-14245 and pBAD18-14255, would allow us to determine if the K279a proteins promoted cell death when they are expressed in the cytoplasm of the heterologous bacterium. By using primer pairs MN76 and MN59 and MN77 and MN61 (Table S2), the two S. maltophilia genes were cloned into pBAD18 without their start codon and instead aligned with the periplasmic localization sequence (ATGAAATACCTGCTGCCGACCGCTGCTGCTGGTCTGCTGCTCCTCGCTGCCCAGCCGGCGATGGCC) of PelB, a well-studied periplasmic protein (120). Once transformed into target E. coli, these constructs, designated pBAD18-pelB14245 and pBAD18-pelB14255, would allow us to determine if the K279a proteins promoted killing when they are expressed in the periplasm of the bacterial competitor. pBAD18-14245, pBAD18-pelB14245, pBAD18-14255, and pBAD18-pelB14255, along with the control pBAD18 vector, were separately transformed into E. coli DH5α. Following growth overnight at 37°C on LB plates containing 100 μg/ml ampicillin, each of the selected clones was resuspended in LB broth to an OD_600_ of 0.3, further diluted in LB broth in 10-fold increments, and then spotted, as 10-μl aliquots, onto ampicillin-containing LB agar with and without 0.4% arabinose to induce the pBRA promoter. Bacterial survival and growth within the spots were visually observed after 18 h of incubation at 37°C. In order to also assess the effect of the cloned S. maltophilia proteins on E. coli in liquid cultures, the various clones were resuspended to an OD_600_ of 0.1 in LB broth with and without arabinose and then allowed to grow for 6 h at 37°C with shaking at 200 rpm (C25KC incubator; New Brunswick Scientific). Once every hour, bacterial survival and growth were assessed by measuring the OD_600_ of the culture and by determining CFU by plating. To determine the protective nature of the cognate immunity protein of 14245 in this assay, a plasmid encoding the immunity protein 14240 (see above) was transformed into E. coli DH5α clones harboring either pBAD18-pelB14245 or the control pBAD18 vector. Following growth overnight at 37°C on LB plates containing 100 μg/ml ampicillin and 50 μg/ml kanamycin, both clones were resuspended in LB broth to an OD_600_ of 0.3 and dilution plated in 10-μl aliquots as described above onto ampicillin- and kanamycin-containing LB agar with 0.4% arabinose to induce expression of pelB14245. Bacterial survival and growth within the spots were visually observed after 18 h of incubation at 37°C.

### Statistical procedures.

In all experiments, each sample or condition was assessed using at least three technical replicates. All experiments were repeated at least three times. The resultant values obtained were presented as the means and standard deviations from the three independent experiments, and statistical analysis was applied using the Student *t* test, as appropriate. *P* values are presented in the figure legends.
