# Supplementary material for: Effectors of the Stenotrophomonas maltophilia Type IV Secretion System Mediate Killing of Clinical Isolates of Pseudomonas aeruginosa
Source: mBio. 2021 Jun 29;12(3):e01502-21. doi: 10.1128/mBio.01502-21 (PMC8262851; doi:10.1128/mBio.01502-21)
Supplement: TABLE S1 [file mbio.01502-21-st001.pdf]

Table S1

A

Strain		Percent Identity to Putative T4SS Effector Proteins of *S. maltophilia* strain K279a												
**Name**	**Source**	**RS00510**	**RS00905**	**RS01275**	**RS01575**	**RS02375**	**RS02385**	**RS02400**	**RS14245**	**RS14255**	**RS14405**	**RS17170**	**RS19100**	**RS20845**
K279a	environmental	100	100	100	100	100	100	100	100	100	100	100	100	100
FDAARGOS_325	clinical	98	99	99	100	50	96	95	92	99	93	99	100	99
CSM2	environmental	92	91	68	43	50	97	83	90	90	86	81	79	30
FDAARGOS_92	clinical	98	98	99	47	49	97	59	100	99	93	75	35	99
SJTH1	environmental	92	95	97	81	97	97	92	92	97	49	82	35	28
FDAARGOS_507	clinical	99	98	99	43	50	96	95	100	100	99	100	35	97
X28	environmental	96	96	98	44	97	97	90	93	95	49	100	35	40
FDAARGOS_649	clinical	98	99	100	47	50	97	58	100	99	93	100	35	99
Sm53	environmental	98	99	99	39	49	96	58	100	99	93	100	100	98
ICU331	environmental	100	100	100	100	50	96	59	100	100	94	100	35	99
NEB515	environmental	98	98	100	42	49	96	93	100	99	98	100	35	98
CF13	clinical	98	97	99	44	50	97	92	100	100	99	100	35	97
NCTC10258	clinical	99	100	99	39	49	96	94	100	100	99	100	100	98
NCTC10498	clinical	99	98	100	39	49	97	58	100	99	93	100	99	100
R551-3	clinical	91	96		44	43	96	91	91	92	46	100	35	92
JV3	environmental	91	91		44	51	96	86	90	90	93	96	96	30
OUC_Est10	environmental	92	95	98	82	97	96	93	92	93	49	80	35	
W18	environmental		96	98	45	50	98	94	91	96	93	100	35	31
KMM 349	environmental	90	95		42	50	96	91	91	90	93	81	78	90
sm454	environmental		99	100	39	49	100	100	100	99	100	100	100	99
sm-RA9	unknown	90	95		42	50	97	92	91	92	93	81	78	91
PEG-42	environmental		96	98	45	50	98	84	91	90	93	100	35	30
NCTC10498	clinical	100	99	99	40	50	96	67	100	99	93		35	99
NCTC10257	clinical	99	99	99	47	49	97	94	99	98	98	100	34	
NCTC13014	environmental		96	98	44	51	97	96	93	89	93	100	35	30
D457	unknown	90	92		45	50	96	96	90	90	85	81	36	
AB550	environmental	91	92		83	98	95	92	90	91	85	80	36	
PEG-68	environmental	90	92		45	48	96	95	90	90	86	81	35	
PEG-390	environmental		95		46	68	97	95	88	89	56	81	37	49
PEG-173	environmental	90	96		43	50	96	91	93	95	86		36	28
CPBW01	environmental		96		45	72	98	94	97	94	57	82	35	30
MER1	environmental		72		80	84	87	64	74	80	74	68	36	88
NCTC10259	environmental	91	92		80	97	94	95	95	90	85	66	35	
ISMMS2	clinical		96		44	98	96	90	92	89	49	82	35	
ISMMS2R	clinical		96		44	98	96	90	92	89	49	82	35	
SKK55	environmental		35		40	72	96	97	90	90	58	82	79	
PEG-305	environmental		38		43	50	95	86	90	89	92	73	78	
PEG-141	environmental		96		82	50	96	91	92	89	92	81	35	
U5	environmental		73		48	49	87	73	74	80	72		79	
AA1*	environmental													
SJTL3*	environmental													
ISMMS3*	clinical													
	**AVERAGE**	**63**	**92**	**54**	**53**	**62**	**96**	**86**	**93**	**93**	**80**	**80**	**52**	**49**

* Strains lacking *virB/D4* T4SS apparatus genes

B

Strain		Percent Identity to Putative Cognate Immunity Proteins of *S. maltophilia* strain K279a												
**Name**	**Source**	**RS00515**	**RS00910**	**RS01280**	**RS01580**	**RS02380**	**RS02390**	**RS02405**	**RS14240**	**RS14260**	**RS14410**	**RS17165**	**RS19095**	**RS20840**
K279a	clinical	100	100	100	100	100	100	100	100	100	100	43	100	100
FDAARGOS_325	clinical	99	100	99	100	43	98	96	78	100	80	100	99	100
Sm53	environmental	99	99	99		42	98	97	100	99	80	95	99	99
sm454	environmental	98	100	98		41	98	99	100	100	99	100	99	100
ICU331	environmental	100	100	100	100	42	98	97	100	100	80	100		99
NCTC10258	clinical	100	100	99		41	98	99	100	100	99	100	100	98
NCTC10498	clinical	97	100	99		42	98	100	100	100	80	98	100	100
D457	unknown	83	93		75	45	97	89	78	80	84	44	57	
OUC_Est10	environmental	86	95	94	75	96	98	90	78	79	78	45		
SJTH1	environmental	85	95	95	76	96	98	93	78	98	94			44
FDAARGOS_507	clinical	98	99	99		97	98	96	100	100	99	98		99
FDAARGOS_649	clinical	98	100	98		42	98	97	100	100	80	100		100
KMM 349	environmental	86	95			46	97	93	77	79	94	42	58	93
sm-RA9	unknown	86	95			46	97	94	77	79	95	43	58	95
NEB515	environmental	97	100	99		40	98	97	100	100	98	100		100
CF13	clinical	99	99	100		42	97	99	100	100	99	100		100
NCTC10257	clinical	100	100	99		41	98	98	100	100	99	97		100
R551-3	environmental	79	94			46	97	94	77	98	95	100		94
JV3	environmental	85	93			44	98	91	87	80	94	91	96	
CSM2	environmental	85	91			45	98	91	87	97	84	45	58	
FDAARGOS_92	clinical	97	99	98		43	97	97	100	99	80			99
X28	environmental	71	97	98		97	98	77	81	98	41	95		
PEG-68	environmental	83	93			94	98	89	78	80	79	44	58	
PEG-42	environmental		96	94		43	98	96	78	79	97	90		40
NCTC10498	clinical	99	100	99		42	98	97	100	100	80			100
NCTC13014	environmental		96	94		42	98	95	88	79	97	99		40
AB550	environmental	83	93		73	97	98	91	78	80	84			
W18	environmental		95	94		42	97	95	79	93	96	90		
U5	environmental		60		72	41	92	82	67	96	81		63	
PEG-173	environmental	85	96			44	99	78	88	99	78			43
PEG-141	environmental		96		79	45	99	78	80	79	97	42		
MER1	environmental		57		71	82	93	85	67	96	76			92
NCTC10259	environmental	83	93		74	97	97	90	96	80	84			
ISMMS2	clinical	95				96	98	78	79	79	41	45		
ISMMS2R	clinical	95				96	98	78	79	79	41	45		
PEG-305	environmental					42	96	92	86	80	98		56	
CPBW01	environmental		95				98	78	98	96		44		40
SKK55	environmental						98	89	86	79		44	56	
PEG-390	environmental		96				99	89	86	80		45		
AA1*	environmental					52	84				32			
SJTL3*	environmental						96	79				97		
ISMMS3*	clinical						96	79						
	**AVERAGE**	**63**	**79**	**47**	**21**	**52**	**97**	**89**	**81**	**84**	**74**	**55**	**28**	**47**

* Strains lacking *virB/D4* T4SS apparatus genes
