# Supplementary material for: Effectors of the Stenotrophomonas maltophilia Type IV Secretion System Mediate Killing of Clinical Isolates of Pseudomonas aeruginosa
Source: mBio. 2021 Jun 29;12(3):e01502-21. doi: 10.1128/mBio.01502-21 (PMC8262851; doi:10.1128/mBio.01502-21)
Supplement: TABLE S2 [file mbio.01502-21-st002.pdf]

# Table S2. Primers used in this study

| Primer Name | 5' to 3' Sequence | Gene |
|---|---|---|
| MN34 | CGCAGTGCAGAAGAGATTGA | RS20845 |
| MN35 | CAGCATGAACGTACCGAAGT | RS20845 |
| MN36 | GATCAGGACCTCGCATGTT | RS19100 |
| MN37 | CGACTGATCTACGGGTGTATTG | RS19100 |
| MN38 | CAGACCTGGCACTGAATCTT | RS14405 |
| MN39 | CAGCAGTTGGGATCGAGTT | RS14405 |
| MN40 | AAACCTGGCTGAGTTGATGT | RS14255 |
| MN41 | CGGCACGCTCGTAATTCT | RS14255 |
| MN42 | GAACCCGGCCAAGAAGAAA | RS14245 |
| MN43 | TGATAGACGGTGCCCTGATA | RS14245 |
| MN44 | TGGAGCAATCGATGCAGAAG | RS02400 |
| MN45 | CGCAGGATGCTGCATTTC | RS02400 |
| MN46 | AAATCACCGAGGAGCACTAC | RS02385 |
| MN47 | CCAGACGAAATCACTGCAAC | RS02385 |
| MN48 | ATTCGTGTCCTGTCCTCCTA | RS02375 |
| MN49 | CAGCAGTCCATAGCCATCATTA | RS02375 |
| MN50 | TCAGATCGCTGAGGTGTTTAAG | RS01575 |
| MN51 | CGTAGAAGTCGCCGTAGTAAAG | RS01575 |
| MN52 | GCAGAAGGTGCTGGAAGAA | RS01275 |
| MN53 | TCCTTGTTGTTGGCGTAGG | RS01275 |
| MN54 | ATCGGTTCCGATGCCAAA | RS00905 |
| MN55 | CGCGTACTTGTGGATGTAGTAG | RS00905 |
| MN56 | GAATCGCTGGATCGCTACAT | RS00510 |
| MN57 | ATCCAGCATCGGCTTGAC | RS00510 |
| MN58 | AAAAAAAAGCTTACTACTACGGTCGTGGCGTCTG | RS14245 |
| MN59 | AAAAAATCTAGATCAGGCCTGCAGGCTGCGGGTC | RS14245 |
| MN60 | AAAAAAAAGCTTTTCAGCGTGATCACCAACGGC | RS14255 |
| MN61 | AAAAAATCTAGACTACAGGGATCGAGAACGCTGC | RS14255 |
| MN62 | AAAAAGCTTGGAACCCTGCAATGGAATGCCCATG | RS14410 |
| MN63 | AAATCTAGACACTTGGACCGGGTGCGCGCGATG | RS14410 |
| MN64 | AAAAAGCTTTCGCTCGCAATGGATTGGACGTGTG | RS01280 |
| MN65 | AAATCTAGATCAGTCCAGCAGCTGCAGATACTTC | RS01280 |
| MN66 | AAAAAGCTTGAACCCTCCTTGGAGCTTCGCCATG | RS02405 |
| MN67 | AAATCTAGATCAGTCTGCCGGCCGATGGTCATAG | RS02405 |
| MN68 | AAAAAGCTTGTGCCCATTCAAGGACGCTCGCATG | RS02390 |
| MN69 | AAATCTAGAGTGCCCATTCAAGGACGCTCGCATG | RS02390 |
| MN70 | AAAAAGCTTCATTGGCGCGCGAGATGCTTTCATG | RS01580 |
| MN71 | AAATCTAGATCAGTGCTGACGGATCGATATGTAG | RS01580 |
| MN72 | AAAAAAAAGATCTGTGACCATCACCTCTCAGGACT | RS14245 |
| MN73 | AAAAAAGGTACCTCAGGCCTGCAGGCTGCGGGTC | RS14245 |
| MN74 | AAAAAAAAGATCTGTGTCGACCGATAGAGAGTCGC | RS14255 |
| MN75 | AAAAAAGGTACCCTACAGGGATCGAGAACGCTGC | RS14255 |
| MN76 | AAAAAAGAATTC**ATGAAATACCTGCTGCCGACCGCT GCTGCTGGTCTGCTGCTCCTCGCTGCCCAGCCGGCGAT GGCCACCATCACCTCTCAGGACTACG** | RS14245 |
| MN77 | AAAAAAGAATTC**ATGAAATACCTGCTGCCGACCGCTG CTGCTGGTCTGCTGCTCCTCGCTGCCCAGCCGGCGATGG CCACCATCACCTCTCAGGACTACG** | RS14255 |
| M13F | GTAAAACGACGGCCAG | |
| M13 R | CAGGAAACAGCTATGAC | |

*PelB sequence in bold
